# Phenylpropanoid methyl esterase unlocks catabolism of aromatic biological nitrification inhibitors

**DOI:** 10.1093/ismejo/wraf251

**Published:** 2025-11-13

**Authors:** Andrew Wilson, Elise Van Fossen, Ritu Shrestha, Andrew Frank, Valentine Trotter, Henri Baldino, Brenton Poirier, Young-Mo Kim, William Nelson, Tuesday Simmons, Devin Coleman-Derr, Adam Deutschbauer, Robert Egbert, Joshua Elmore

**Affiliations:** Biological Science Division, Pacific Northwest National Laboratory, Richland, WA 99352, United States; Biological Science Division, Pacific Northwest National Laboratory, Richland, WA 99352, United States; Biological Science Division, Pacific Northwest National Laboratory, Richland, WA 99352, United States; Biological Science Division, Pacific Northwest National Laboratory, Richland, WA 99352, United States; Environmental Genomics and Systems Biology Division, Lawrence Berkley National Laboratory, Berkeley, CA 94720, United States; Biological Science Division, Pacific Northwest National Laboratory, Richland, WA 99352, United States; Biological Science Division, Pacific Northwest National Laboratory, Richland, WA 99352, United States; Biological Science Division, Pacific Northwest National Laboratory, Richland, WA 99352, United States; Biological Science Division, Pacific Northwest National Laboratory, Richland, WA 99352, United States; Plant Gene Expression Center, USDA-ARS, Albany, CA 94710, United States; Plant Gene Expression Center, USDA-ARS, Albany, CA 94710, United States; Environmental Genomics and Systems Biology Division, Lawrence Berkley National Laboratory, Berkeley, CA 94720, United States; Biological Science Division, Pacific Northwest National Laboratory, Richland, WA 99352, United States; Biological Science Division, Pacific Northwest National Laboratory, Richland, WA 99352, United States

**Keywords:** microbiology, microbial genetics, nitrification, metabolic pathways, bacteria, rhizosphere, catabolism, biological nitrification inhibition, bacterial physiology, genetic engineering

## Abstract

Microbial nitrification of fertilizers represents is a significant global source of greenhouse gas emissions. This process increases emissions, fosters toxic algal blooms, and raises crop production costs. Some plants naturally release biological nitrification inhibitors to suppress ammonium-oxidizing microbes and reduce nitrification. Engineering nitrification inhibitor production into food and bioenergy crops via synthetic biology offers a promising mitigation strategy, but its success depends on addressing gaps in our understanding of inhibitor degradation in soil. This study begins to fill this gap by identifying a previously unknown microbial pathway for degrading phenylpropanoid methyl esters, a key class of aromatic nitrification inhibitors. Using transcriptomics and high-throughput functional genomics, we discovered genes essential for phenylpropanoid methyl ester degradation. Genetic and biochemical analyses revealed two novel enzymes, including a newly identified phenylpropanoid methyl esterase, that direct phenylpropanoid methyl esters into known metabolic pathways. Importantly, transferring these genes into bacteria capable of metabolizing other phenylpropanoids enabled them to use the methyl esters as a carbon source. This work provides critical insights into microbial nitrification inhibitor degradation, a poorly understood element of the nitrification cycle.

## Introduction

**Figure 1:**
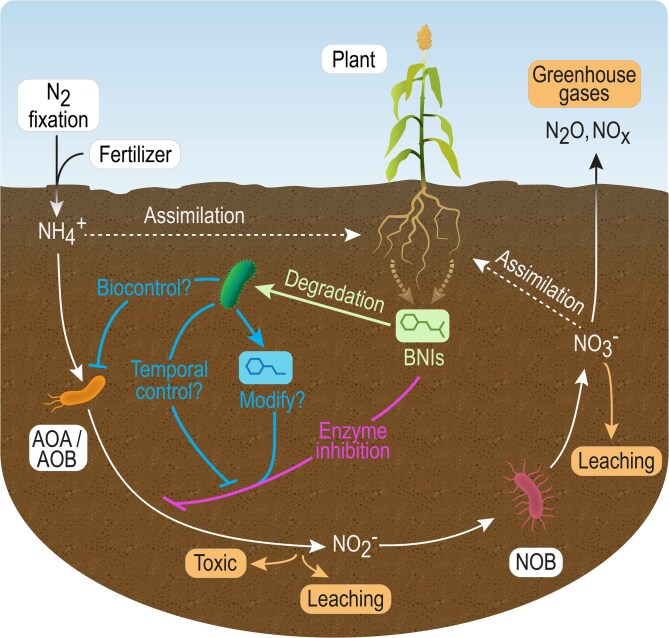
Biological inhibition of the nitrification cycle. AOA, AOB, and nitrite oxidizing bacteria convert excess N in the soil into forms that leach into soils and are released as nitrogenous GHGs. Plant roots exude BNI chemicals to prevent loss of soil nitrogen. The primary known BNI mode-of-action is the inhibition of ammonia oxidizing enzymes. A more complete understanding of the nitrogen cycle requires investigation of microbes capable of consuming BNIs. Microbial degradation of BNIs has the potential to restore nitrification. In contrast, some BNIs may function as growth substrates for microbes that interfere with AOA/AOB growth (i.e. biocontrol).

Agriculture is a major source of greenhouse gas (GHG) emissions [1]. Although application of N-fertilizers increases crop yields [2], a large proportion of applied N-fertilizer is ultimately lost to the atmosphere as N_2_O, a potent GHG (Fig. 1) [3, 4]. Nitrifying microbes catalyze the oxidation of reduced forms of nitrogen (NH_3,_ NH_4_^+^) into nitrate (NO_3_^−^) and NO_3_^−^ is converted to the potent GHG N_2_O by denitrifying bacteria that are widely prevalent in soil [5]. One approach to mitigate these losses is to limit the application of chemical fertilizers. Engineered microbial and plant [6] systems can be deployed in agricultural soils to enhance fixation of elemental nitrogen (N_2_) into biologically available nitrogen (NH_3_) [6–11]. Supplementing soils with engineered N_2_-fixing bacteria can substantially reduce chemical N-fertilizer requirements for robust crop yields and thus indirectly address nitrification.

A second approach to reduce nitrification in agriculture is the co-application of chemical or biological nitrification inhibitors (BNIs) with fertilizers [12, 13]. Though the inhibition mechanisms of these compounds are not generally understood, their application prevents growth of ammonia oxidizing archaea (AOA) and bacteria (AOB). Canonically, nitrification inhibitors are thought to inhibit activity of the highly conserved ammonium oxidase (AMO) and hydroxylamine oxidoreductase (HAO) nitrification enzymes present in limited AOA and AOB lineages. Purified BNIs can be applied exogenously or delivered into the soil directly by plants, or potentially engineered microbes, to improve plant nitrogen use efficiency [14]. Many plants natively secrete BNI compounds and an increasing number of plants—including rice, maize, and sorghum [15–18]—have been found to produce chemically distinct BNI. These BNI compounds include phenolics, lipophilic benzoquinones, terpenes, benzoxazinoids, and diols [16–18]. Advances in synthetic biology have enabled microbe and plant engineering to produce chemicals similar to BNIs (e.g. *p-*coumarate, terpenes) [19–21]. An improved understanding of BNI modes-of-action and mechanisms of microbial BNI degradation will likely advance nitrification reduction by guiding strategies for engineering BNI processes and help determine effective approaches to apply BNI.

Current methods to identify BNIs fall short of mechanistic understanding to drive engineering solutions. Measurements of ammonium and nitrate in soils supplemented with BNI compounds [22], indirect whole cell *in vitro* assays [23], and microbial abundance assays (e.g. 16s rRNA gene sequencing) do not provide direct mechanistic evidence of the processes driving biological nitrification inhibition [22]. The indirect nature of these nitrification assays can lead to incorrect conclusions that BNI compounds function exclusively through inhibition of AMO or HAO enzyme activity rather than disrupting other cellular functions. The chemical diversity of BNI compounds and knowledge that these compounds have other functions in the environment [24, 25] (e.g. herbicidal activity of the BNI sorgoleone [25]) suggests that BNI compounds may have additional physiological functions or may target other critical enzymes produced by AOA/AOB [17]. Plants respond to stress conditions by exuding compounds into the rhizosphere that encourage growth of microbes with beneficial functions (e.g. N-fixation, antifungal activity) [26–28]. Similarly, exuded BNIs may perform secondary functions, such as encouraging growth of microbes that directly or indirectly inhibit growth of ammonium oxidizing microbes (Fig. 1).

Another key challenge in understanding nitrification is our limited knowledge of BNI degradation, which limits predictions of the efficacy of specific BNIs in a soil ecosystem. BNIs are subject to mineralization in the environment and their degradation dynamics have been observed to vary across ecosystems [29]. For example, genes that encode enzymes involved in the degradation of sorgoleone are abundant in *Streptomyces* strains isolated from the rhizosphere of *Sorghum bicolor*, but are rare in related strains from the rhizosphere of *Populus trichocarpa* [30]. Although the presence of such genes in a metagenome could provide useful insights for applying specific BNIs, the enzymes responsible for BNI degradation remain largely unidentified.

In this study, we advance the understanding of BNI degradation by identifying two environmental bacteria that catabolize phenylpropanoid methyl esters (PPMEs)—a class of phenolic BNI produced by multiple plants [15, 24, 31]—for growth. PPME act as biological nitrification inhibitors [15, 31]. However, their mechanisms of action and their production and degradation processes remain poorly understood. Pseudomonads are ubiquitous in soil and rhizosphere environments where they frequently encounter phenylpropanoids such as *p-*coumarate in plant root exudates and decomposing plant biomass. Many environmental pseudomonads use phenylpropanoids as carbon sources to support growth [32–34], which led us to investigate their ability to utilize PPMEs as growth substrates.

Using a combination of genome-wide transposon mutagenesis fitness assays and transcriptomics, we discovered and characterized a metabolic pathway that is responsible for PPME degradation. Additionally, we identified a novel enzyme that performs the initial step in PPME catabolism. This step permits PPMEs to flow into a known pathway for phenylpropanoid metabolism. Finally, we transferred this metabolic pathway into multiple soil bacteria to demonstrate heterologous PPME catabolism. Our findings uncover the molecular mechanism behind PPME degradation, provide functional annotations for genes encoding the enzymes involved, and demonstrate the capacity for broad adoption of PPME catabolism by pseudomonads through horizontal gene transfer. These annotations will pave the way for sequencing-based methods to predict a microbiome’s ability to rapidly degrade this class of BNIs.

## Materials and methods

### General culture conditions & media

Strains and plasmids used are listed in Supplementary Table S1. Routine cultivation of *Escherichia coli* for plasmid construction and maintenance was performed at 37°C using LB medium supplemented with 50 *μ*g/ml kanamycin sulfate or 50 *μ*g/ml apramycin sulfate and 15 g/l agar (for solid medium). Routine cultivations for all *Pseudomonas* strains were performed at 30°C with 200 rpm shaking. These routine cultivations were performed using in LB medium for all strains other than TBS10 and Super Optimal Broth (SOB) [35] for TBS10. The defined mineral medium MME (MOPS medium enhanced) was used for cultivation assays. MME is buffered by 20 mM MOPS (3-(N-morpholino)propanesulfonic acid) to a pH of 7, lacks carbon sources, and includes ammonium chloride as a nitrogen source. The specific composition of MME has been described in detail previously [36]. Other than glucose, all carbon sources were used at 2.5 mM. Glucose was used at either 10 mM (growth assays and RNAseq cultures) or 5 mM (fitness assays).

### Plasmid construction

PCR amplification for plasmid construction used Q5 DNA polymerase (NEB) and Eurofins Genomics primers. Colony PCR used OneTaq (NEB). Plasmids were built via using NEBuilder HiFi DNA Assembly Master Mix (NEB) or using T4 DNA ligase (NEB) and transformed into NEB 5-alpha F’I^Q^ using standard *E. coli* protocols. PCR template DNA from *Pseudomonas fluorescens* SBW25 or *Pseudomonas* sp. TBS28 was extracted using using Zymo Quick gDNA miniprep kit (Zymo Research). Gel extractions and PCR purifications used Zymoclean Gel DNA recovery kit (Zymo Research), plasmid preps used GeneJet plasmid miniprep (ThermoScientific) or ZymoPURE kits (Zymo Research), and sequences were verified via Sanger sequencing (Eurofins). Plasmids are in Supplementary Table S1 and DNA maps in Supplementary File F1.

### Pseudomonad strain construction

Genome edits used allelic exchange (gene deletions and poly-*attB* cassette insertion) or integration of nonreplicating plasmids (expression cassette insertion) with methods described previously [36–39]. Plasmids for expressing PFLU3311, PFLU3296, or PFA28_2292 were integrated into host chromosomes using Bxb1 integrase. Briefly, Bxb1 integrase unidirectionally recombines DNA between a cognate *attB* and *attP* sequences. Expression plasmids each harbor a Bxb1 *attP* sequence, and the engineered variants of *Pseudomonas putida* KT2440 (JE90), *P. fluorescens* SBW25 (JE4621), *Pseudomonas fredericksbergensis* TBS10 (JE5041), and *Pseudomonas* sp. TBS28 (RS175) used here contain a Bxb1 *attB* sequence. Strains JE90, JE4621, and JE5041 were produced previously [38, 39]. A poly-attB site containing 10 distinct *attB* sequences was integrated downstream of *ampC* (PFA28_1309) to generate RS175. Strain construction was verified using colony PCR. Mutant strains and the plasmids used to generate them are listed in Supplementary Table S1, respectively. Primers are listed in Supplementary Table S2.

### Plate reader growth assays

Freezer stocks were revived in SOB or LB medium, then inoculated into MME with 10 mM glucose for starter cultures. These carbon-limited cultures were incubated (30°C, 200 rpm) to stationary phase, ensuring normalized inoculum and no residual carbon source. Growth assays were performed in clear 48-well plates (Greiner Bio-One) containing 600 *μ*L MME + carbon sources in each well. Plates were incubated at 30°C, 548 rpm in BioTek Neo2SM or Synergy H1 plate readers with OD600 readings taken every 10 min. Plots are representative curves from three biological replicates.

### Randomly barcoded transposon sequencing fitness assays

Randomly barcoded transposon sequencing (RB-TnSeq) is a method used to measure the abundance of strains with transposon insertions and assess gene fitness in a competitive growth assay [40]. To evaluate the fitness of a gene under specific experimental conditions, the cumulative barcode counts for strains associated with that gene (independent transposon insertions) are compared before and after growth selection. A fitness score is determined by the log_2_ fold-change in cumulative mutant abundance during growth (typically lasting 6 to 8 generations) as determined by barcode sequencing. The fitness score of a gene is used to infer how important the gene is for growth in the experimental condition.

RB-TnSeq was performed and analyzed according to the protocols described previously [40]. Briefly, glycerol stocks of mutagenized *P. fluorescens* JE4621 libraries were recovered by cultivation in LB media to an OD_600_ of 0.5. The resulting mutant library was adapted to the defined MME by cultivation in an MME variant containing 5 mM glucose, 5 mM citrate, and 15 mM acetate as the growth substrates. Adaptation enables minimizes changes in mutant abundance that are associated transition from complex LB medium to defined MME medium. The adapted cells were washed in MME without a growth substrate to remove residual growth substrates from the prior cultivation and resuspended to an OD_600_ of 2. Aliquots of washed cells were stored at −80°C as T = 0 samples. Competitive growth assays were performed by inoculating MME containing various carbon sources with washed cells to an OD_600_ of 0.02 followed by cultivation in 24-well plates at 30°C, 700 rpm (2 mM orbital) until stationary phase. Cells were harvested for amplicon sequencing and barcode quantification as described previously [40]. Fitness data are in Supplementary File F1 and barcoded transposon insertion sites are in Supplementary File F3. Code for analyzing RB-TnSeq data is available at https://bitbucket.org/berkeleylab/feba. Raw sequence data and processed fitness values are available from http://genomics.lbl.gov/supplemental/rbarseq/ along with scripts for reproducing all of our results.

### Transcriptomics experiments and differential expression analysis

*P. fluorescens* SBW25 was cultured at 30°C in 50 ml MME medium supplemented with either 10 mM glucose or 5 mM MHPP (methyl 3-(4-hydroxyphenyl)propionate) in a 250 ml Erlenmeyer shake flask at 30°C, 200 rpm shaking and harvested mid-exponential growth phase (OD_600_ = ~0.2) by centrifugation (~16 000 × g, 2 min, 4°C). Supernatants were quickly decanted, and cell pellets were frozen rapidly in liquid nitrogen prior to storage at −80°C. Four samples were prepared for each condition. Cell pellets were submitted to the company GeneWiz, a subsidiary of Azenta Life Sciences, for RNA extraction, ribosomal RNA depletion and RNA sequencing.

After Illumina sequencing, RNA-seq reads were mapped to the *P. fluorescens* SBW25 reference genome (NC_012660) using the Geneious for RNA-seq mapping workflow in the Geneious Prime software package. Read count per annotated gene was calculated for each treatment and replicate, as well as fragment per kilobase million, a common normalization technique. We then used the implementation of the DEseq2 R-package that is built into Geneious to calculate differential gene expression. DESeq2 calculates log-fold change in expression and allows comparison between treatments [41]. We had four replicates per treatment. Differential expression data, DEseq2 output, can be found in Supplementary File F2. RNAseq data were deposited in the Gene Expression Omnibus under accession number GSE228022.

### Phylogenetic analysis of protein sequences

Protein sequences from functionally validated enzymes and other representative uncharacterized proteins were obtained from GenBank. Protein sequences were aligned through the Aliview (v1.26) alignment viewer and editor [42] using mafft (v7.453) [43] with the –localpair option and a maximum of 1000 iterations. The tree was constructed using FastTree [44] and visualized using FigTree (http://tree.bio.ed.ac.uk/software/figtree/).

### Recombinant expression and protein purification

Recombinant Pme (PFLU3311) protein expression and purification performed was using standard Immobilized Metal Affinity Chromatography workflows as described previously [45] with the following modifications. Cultures for protein expression were grown using *E. coli* BL21(DE3) colonies harboring pEVF-SBP1-P2, an expression vector for His_6_-tagged Pme. Protein concentration was determined by absorbance at 280 nm and flash-frozen with liquid nitrogen and stored at −80°C until needed.

### Methyl esterase enzyme assays

Enzyme activity assays were conducted in triplicate under two conditions: with and without the addition of purified enzyme (500 nM). Each reaction mixture was prepared using 500 *μ*L of 25 mM ammonium bicarbonate buffer (pH 7.5), supplemented with one of the substrates—MHPP, methyl trans-p-coumarate, methyl cis-p-coumarate, or methyl ferulate—at a final concentration of 5 mM. The samples were incubated at 25°C for 1 h. To terminate the reactions, a 3-kDa MWCO Vivaspin concentrator was used to separate proteins from the reaction mixtures. The resulting filtrates were diluted 100-fold in distilled water for subsequent analysis via liquid chromatography-mass spectrometry (LC–MS).

### Liquid chromatography—mass spectrometry analysis of enzyme assay products

Ten microliters of sample was injected, and analytes were separated on a ZORBAX Extend C18 Column (2.1 × 150 mm; 1.8 *μ*m) maintained at 24°C with a flow rate of 300 *μ*L min^−1^. The mobile phase, comprising water with 0.1% formic acid (A) and acetonitrile with 0.1% formic acid (B) was held at 95% A for the first 1.12 min, ramped to 99.5% B at 6.4 min, and held at 99.5% until 10 min. The mobile phase was held at 95% A for 2 min following data acquisition for post-run conditioning. Data were acquired with a Waters Xevo G2 QTof using positive ionization to detect ferulate, methyl ferulate, and MHPP, and negative ionization for *p*-coumarate, methyl coumarate, and phloretate. Conditions in the spray chamber were as follows: 80°C source temperature; 150°C desolvation temperature; 600 L h^−1^ desolvation gas flow; and 40 V cone voltage. A capillary voltage of 2.5 kV was used, and mass spectra were continuously recorded within a m/z 50–1200 range. Compounds were identified by comparison with authentic standards and chromatograms were displayed with base peak intensity (BPI), or total ion current.

## Results

### Select environmental bacteria can use phenylpropanoid methyl esters as a growth substrate

We initially evaluated the ability of multiple environmental pseudomonads to grow PPMEs as growth substrates. These included four well studied strains: *P. fluorescens* SBW25 [46], *P. putida* KT2440 [47], *Pseudomonas protegens* Pf-5 [48], and *Pseudomonas stutzeri* DSM4166 [49]. We also evaluated three recently isolated endophytes from *S. bicolor* [50], a plant known to produce multiple BNIs, including methyl 3-(4-hydroxyphenyl) propionate (MHPP)—the first PPME identified as a nitrification inhibitor [15]. Strain TBS10 was previously classified as *Pseudomonas frederiksbergensis* [39], but strains TBS28 and TBS49 lacked sufficient similarity with existing species to assign species-level classification [50]. TBS49 is related to the *P. putida* group, and we refer to it as *Pseudomonas* sp. TBS49. Using the Genome Taxonomy Database [51], we identified strain TBS28 as a member of a currently unnamed *Pseudomonas* species. For simplicity, we will refer to each strain by its identifier (e.g. TBS28).

**Figure 2:**
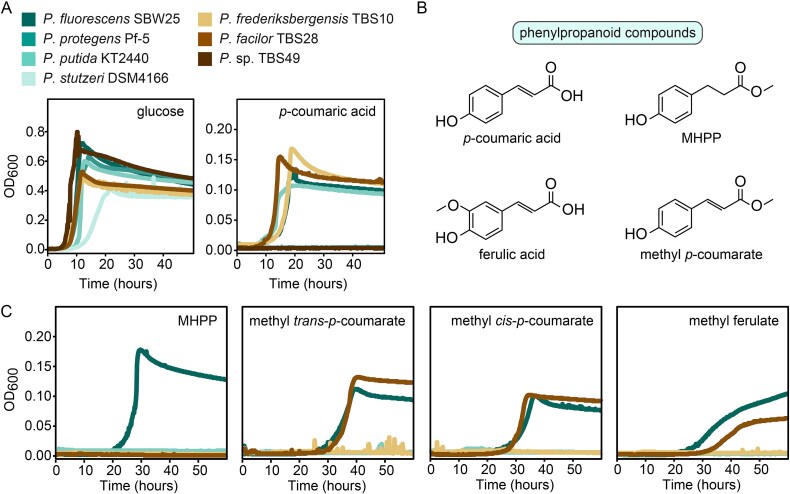
A subset of environmental Pseudomonas isolates can utilize PPMEs as carbon sources. Microtiter plate cultivation assays comparing growth of environmental pseudomonads using (A) two control carbon sources and (C) four PPMEs. Structures of phenylpropanoids are shown in panel (B). Experiments in panel C only include the four pseudomonads that grew with *p-*coumaric acid in panel a. assays were performed with MME medium containing either 10 mM (glucose) or 2.5 mM (all others) of the indicated carbon sources. Each panel contains a single representative curve from one of three biological replicates.

We evaluated whether each strain could grow on glucose or *p-*coumarate, a model phenylpropanoid, as sole growth substrates to investigate carbon utilization capabilities. As expected, each strain grew robustly with glucose, but only *P. putida*, *P. fluorescens*, *P. frederiksbergensis*, and TBS28 were able to consume *p-*coumarate (Fig. 2A). Genome analysis revealed that these four strains possess genes encoding a known pathway for *p-*coumarate degradation (Supplementary Fig. S1). The phenylpropanoid *p*-coumarate is commonly detected in plant root exudates and shares structural similarity to PPMEs (Fig. 2B), and thus the capacity to degrade *p-*coumarate could indicate potential for PPME metabolism.

To further assess PPME utilization, we evaluated these four strains for their ability to grow using four distinct PPMEs as growth substrates, as well as the related phenylpropanoid phloretate (Fig. 2C, Supplementary Fig. S2). The PPMEs tested include methyl *p*-coumarate, methyl ferulate, and MHPP. Phloretate is a potential intermediate of MHPP catabolism and thus the ability to utilize it as a growth substrate was tested as well. Among the strains, *P. fluorescens* could use all tested compounds as growth substrates. Although TBS28 used two of the PPMEs, it could not consume MHPP or phloretate. *P. frederiksbergensis* and *P. putida* were unable to use any of the PPMEs, but *P. putida* was able to utilize phloretate as a growth substrate.

Given its ability to consume all tested PPMEs and phloretate, *P. fluorescens* was selected as a model to investigate the metabolic pathway underlying PPME degradation. Established genetics for this strain provides a testbed to elucidate how these compounds are processed in microbial communities and how they influence nitrification inhibition and microbial coexistence in soil ecosystems.

### Genome-wide fitness assays in *P. fluorescens* reveal critical genes for use of methyl 3-(4-hydroxyphenyl) propionate as a growth substrate

To identify genes involved in catabolism of PPMEs, we employed high-throughput RB-TnSeq [40]. This method enables the quantification of each gene’s contribution to fitness in tested conditions by measuring changes in the relative abundance of genetically barcoded gene disruption mutants within a pooled population. Genes with large negative fitness values (< −1.8), or low fitness scores, are generally important for growth in the test condition [40, 52, 53], but genes with large positive fitness values (>1.8), or high fitness scores, indicate that elimination of the encoded protein improves growth [54, 55]. For this study, we generated a barcoded transposon mutant library in *P. fluorescens* JE4621, which is a SBW25-derivative containing a genome integrated poly-*attB* cassette that enables use of serine recombinase-assisted genome engineering [39]—a tool we routinely utilize for genetic manipulation of bacteria.

We profiled the RB-TnSeq mutant library to assess fitness during growth on PPMEs, phenylpropanoids, and control carbon sources. These include MHPP, ferulate, *p*-coumarate, 4-hydroxybenzoate (an intermediate in degradation of *p-*coumarate), and glucose (a structurally unrelated carbon source). Between 82 (glucose) and 271 (MHPP) genes were identified with low fitness scores across the conditions (Supplementary File F1). When MHPP was used as a carbon source, we observed far more genes (271 genes) with low fitness scores than on any other carbon source (82 to 132 genes). With the exception of MHPP (56 genes), genes with high fitness scores were relatively rare (0 to 18 genes) for all carbon sources.

To refine a target set of genes critical for PPME catabolism, we integrated the fitness data from RB-TnSeq with expression data from RNAseq experiments. Typically, RB-TnSeq identifies a small subset of genes that are uniquely involved in catabolism of a specific carbon source by comparing fitness values across multiple growth substrates. For MHPP, the large number of genes that influenced fitness during growth made it impractical to individually evaluate each gene for roles in MHPP catabolism. These results suggest that PPMEs may have physiological effects beyond providing a growth substrate, such as introducing toxic products from metabolism or inducing regulatory impacts on cellular physiology. By combining genome-wide fitness assays with differential transcriptomics, we aimed to isolate genes specifically involved in PPME catabolism from those related to broader cellular responses to the compounds.

### Combining randomly barcoded transposon sequencing and RNAseq refines candidate genes for phenylpropanoid methyl ester catabolism

To identify highly expressed genes during MHPP consumption, we performed RNAseq on *P. fluorescens* cells grown on glucose or MHPP as the growth substrate. A total of 43 genes were upregulated by at least 8-fold in MHPP-grown cultures compared to glucose-grown cultures (Supplementary File F2, Supplementary Fig. S3). Genes in the *ech* gene cluster (PFLU3296-3303), which encode enzymes involved in the initial steps of *p-*coumarate catabolism, were highly expressed when MHPP was the growth substrate (Table 1). In addition, we identified a neighboring cluster of genes (PFLU3304-3311) that was highly expressed in MHPP-grown cells (Table 1, Supplementary Fig. S4b). The *ech* gene cluster is highly conserved among the four *p-*coumarate consuming pseudomonads we evaluated, both in protein sequence (Supplementary Table S3) and gene synteny (Supplementary Fig. S4a). However, the second gene cluster is only partially conserved between *P. fluorescens* and the sorghum isolates and is completely absent in KT2440 (Supplementary Fig. S4b). This cluster includes genes predicted to encode two putative oxidoreductases, a serine hydrolase, multiple transcription factors, several transporters, and two hypothetical proteins.

**Table 1:** Differential expression values and fitness values for genes in the p-coumarate and adjacent gene cluster.

Locus tag	Annotated function	Log2 fold change (MHPP vs. glucose) in expression	Glucose mean fitness	*p*-coumarate mean fitness	Phloretate mean fitness	4-HB mean fitness	Ferulate mean fitness	Methyl *p*-coumarate mean fitness	MHPP mean fitness
PFLU3296 (*pcd*)	Acyl-CoA dehydrogenase	7.47	−0.091	−0.150	−4.475	−0.206	−0.157	−0.202	−5.092
PFLU3297	Thiolase family protein	7.78	−0.058	0.036	−2.962	−0.064	−0.181	0.226	−4.196
PFLU3298 (*fcs*)	Feruloyl-CoA synthase	8.09	0.130	−3.411	−4.670	0.323	−2.061	−2.461	−5.189
PFLU3299 (*vdh*)	Vanillin dehydrogenase	8.40	0.015	−3.936	−5.676	0.252	−2.815	−2.89725	−5.830
PFLU3300 (*ech*)	*p*-hydroxycinnamoyl CoA hydratase/lyase	8.26	−0.097	−3.953	−4.491	−0.084	−3.875	−2.23375	−5.190
PFLU3301^a^	MarR family transcriptional regulator	3.17	—	—	—	—	—	—	—
PFLU3302	OprD family porin	8.31	−0.009	3.421	0.155	0.091	5.020	−0.129	−0.749
PFLU3303 (*mhpT*)	3-(3-hydroxy-phenyl)propionate transporter	7.73	−0.022	0.013	−0.092	−0.013	−0.029	−0.32725	−1.194
PFLU3304	Hypothetical protein	6.52	0.054	−0.153	−0.152	−0.064	0.007	−0.6245	0.216
PFLU3305	SDR family oxidoreductase	5.66	−0.085	−0.154	−0.150	0.061	−0.178	−0.91225	0.282
PFLU3306	Benzaldehyde dehydrogenase	6.99	0.118	−0.095	−0.060	0.213	−0.018	−0.715	0.972
PFLU3307	Sigma-54-dependent Fis family transcriptional regulator	2.52	0.165	0.078	0.054	0.460	0.129	−0.932	0.933
PFLU3308	TetR/AcrR family transcriptional regulator	2.46	0.025	−0.113	−0.034	−0.010	−0.002	−1.57725	−4.288
PFLU3309	Polyamine ABC transporter substrate-binding protein	7.43	−0.060	−0.051	−0.030	−0.120	−0.110	0.33925	0.642
PFLU3310	Hypothetical protein	8.14	0.076	0.066	0.193	0.000	−0.083	1.04625	0.522
PFLU3311 (*pme*)	Serine hydrolase	7.69	0.012	0.047	−0.039	0.006	0.031	−2.27625	2.722

^a^The — symbol indicates that no fitness data available.

**Figure 3:**
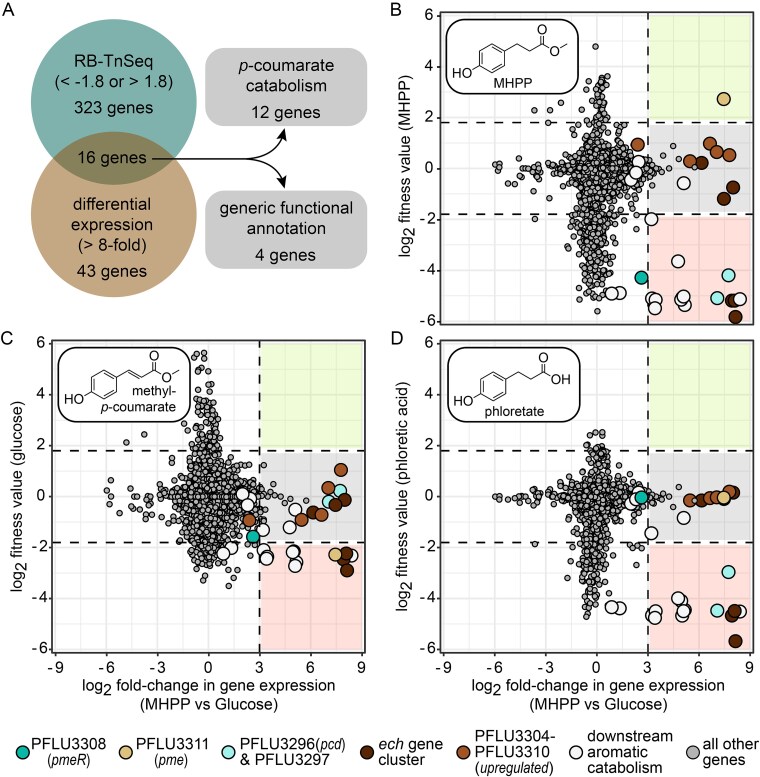
Co-evaluation of genome-wide fitness and differential gene expression narrows search for genes involved in PPME catabolism. (A) Venn diagram showing overlap between genes with large fitness values when SBW25 is grown using MHPP and genes that are more highly expressed when SBW25 is grown with MHPP compared to glucose. (B–D) Plots comparing differential expression (x-axis) versus mean RB-TnSeq fitness values (y-axis), with fitness values from cultures grown with (B) MHPP, (C) methyl *p*-coumarate acid, or (D) phloretate. Positive and negative differential expression values indicate higher expression during growth using MHPP and glucose, respectively. Large dots indicate genes encoding the putative PPME-responsive *pmeR* transcription factor, phenylpropanoid methyl esterase (PFLU3311), putative phloretoyl-CoA dehydrogenase (PFLU3296), putative β-ketothiolase (PFLU3297), other genes in the *ech* gene cluster, other genes in the MHPP-upregulated gene cluster, and downstream aromatic catabolic pathway gene clusters. Small dots represent all other genes. Values represent the mean of four RB-TnSeq or four differential expression biological replicates. Genes lacking fitness or differential expression data are not displayed.

We then co-evaluated RB-TnSeq data with RNAseq results to identify genes directly involved in PPME catabolism. Specifically, we focused on a subset of genes that satisfied two conditions: (i) large positive or negative fitness values in experiments using MHPP as the growth substrate and (ii) significantly higher gene expression in MHPP-grown cultures compared to glucose-grown cultures (Fig. 3, Supplementary Fig. S5). Using these criteria, we narrowed the list to 16 candidate genes. Of these, 12 genes displayed similar fitness values across the model aromatic growth substrates (*p*-coumarate, 4- hydroxybenzoate, and ferulate) and the PPMEs, suggesting they encode enzymes involved in known aromatic degradation pathways (Supplementary Fig. S1). Among these genes are three from the *ech* operon, which encode enzymes that perform the initial steps of *p-*coumarate and ferulate catabolism. The remaining nine genes encode enzymes required for complete conversion of *p*-coumarate and other related aromatics (e.g. 4-hydroxybenzoate) into intermediates of the TCA cycle, including genes encoding all enzymes shown map to known enzymes (Supplementary Fig. S1). Based on these data, we hypothesized that MHPP is converted into an intermediate in the *p*-coumaric catabolic pathway.

The remaining four genes (PFLU3311, PFLU3308, and PFLU3296-3297) exhibited unique fitness effects, significantly influencing growth only when MHPP was used as the growth substrate (Fig. 3B). MHPP differs from *p-*coumarate by its methyl ester moiety and saturated propyl chain. To identify genes responsible for removal of the methyl ester moiety and saturated propyl chain, we performed additional RB-TnSeq experiments using methyl *p-*coumarate and phloretate, respectively (Fig. 3C and D). Among these genes, disruption of PFLU3308, a putative transcription factor, impaired growth on both MHPP and methyl *p-*coumarate but not phloretate, suggesting it has a role in regulating enzymes essential for demethylating PPME substrates. The remaining three genes encode putative enzymes that caused varying substrate-dependent growth defects when disrupted, pointing towards distinct roles in the catabolism of MHPP.

### PFLU3311 encodes a novel phenylpropanoid methyl esterase

**Figure 4:**
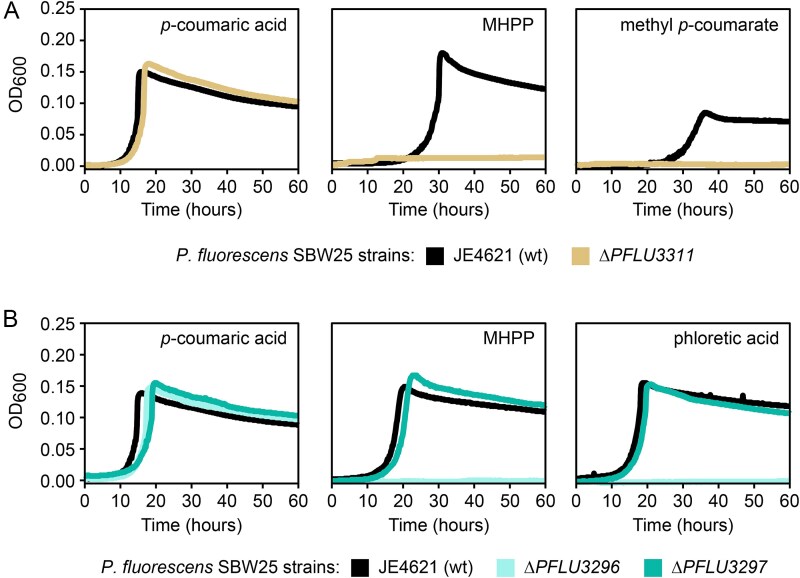
Genetic dissection of a proposed pathway for PPME catabolism. Microtiter plate cultivation comparing growth of select gene deletion mutants *of P. fluorescens* SBW25. Assays were performed with MME medium containing 2.5 mM each of the indicated carbon sources. Each panel contains a representative growth curve from one of three biological replicates.

Disruption of PFLU3311 by Tn insertion influenced growth with the two methyl esters, but not phloretate, suggesting a role in removing the methyl moiety. Although we observed that transposon-based disruption of the gene led to a decrease in fitness with methyl *p*-coumarate, we unexpectedly found that PLFU3311 mutants had a fitness advantage with MHPP. We generated a targeted gene deletion in *P. fluorescens* and compared growth of this mutant with its parent strain using *p*-coumarate, MHPP, and methyl *p*-coumarate as carbon sources to better understand its role in PPME catabolism (Fig. 4A). Even though disruption of PFLU3311 in the RB-TnSeq experiments led to an increase in fitness with MHPP, we observed that the deletion mutant was unable to grow in axenic cultures with MHPP or methyl *p-*coumarate as the sole growth substrate. Incorporation of a constitutively expressed copy of PFLU3311 into mutant strain at a second location in the chromosome restored growth with the PPMEs (Supplementary Fig. S6). Together this suggested that the protein encoded by PFLU3311 is likely responsible for removing the methyl group from PPMEs.

PFLU3311 encodes a putative serine hydrolyase, one of the largest classes of enzymes [56, 57] with functions spanning from proteases [58] to hydrolysis of the ester linkages in polyethylene terephthalate by PETase [59]. The protein encoded by PFLU3311 has low sequence homology with any well characterized enzymes (Supplementary Fig. S7—protein similarity tree), including a functionally similar feruloyl esterase family that is thought to be responsible for breaking the phenylpropanoid-sugar ester linkages found between lignin and hemicellulose [60]. The most similar proteins with an experimentally validated function are two 6-aminohexanoate dimer hydrolases that cleave amide bonds in linear nylon oligomers [61, 62] (Supplementary Table S4). Thus, we hypothesized that the serine hydrolase encoded by PFLU3311 is a phenylpropanoid methyl esterase (Pme) that cleaves the ester bond in MHPP and methyl *p*-coumarate.

**Figure 5:**
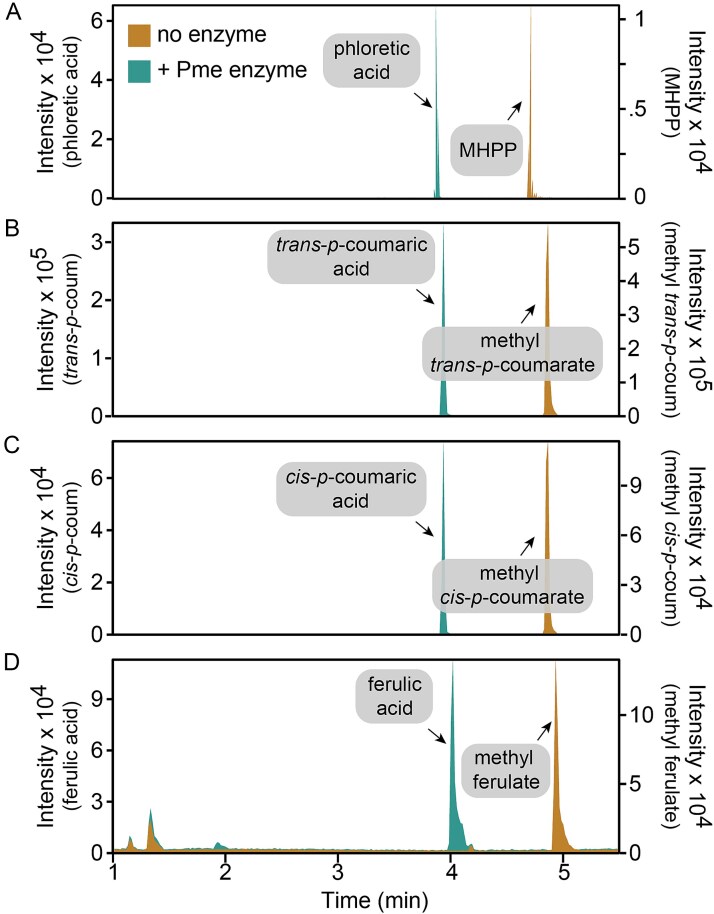
PFLU3311 encodes a phenylpropanoid methyl esterase. Overlayed LC–MS chromatograms of products in enzyme assay samples containing (A) MHPP, (B) methyl-*trans-p-*coumarate, (C) methyl-*cis-p-*coumarate, and (D) methyl ferulate. Chromatograms of samples with and without the protein encoded by PFLU3311 are indicated. For each compound other than methyl ferulate, there is a single predominant ion and thus for panels a-c the intensity is represents BPI. Methyl ferulate had two prominent peaks, and thus intensity represents the total ion count. Each displayed chromatogram is a single representative of 3 replicate enzyme activity assays.

We confirmed the biochemical function PFLU3311 using *in vitro* enzyme activity assays. For this, we expressed and purified a codon-optimized version of PFLU3311 containing a C-terminal 6x-His-tag from *Escherichia coli* BL21(DE3) pLysS, by immobilized metal affinity chromatography. The purified protein was incubated with four PPMEs and the resulting products were analyzed by LC–MS (Fig. 5). Unlike feruloyl esterases, which show poor activity with PPMEs [60], the enzyme encoded by PFLU3311 fully converted each PPME into its cognate carboxylic acid. This combined with the gene’s close proximity to phenylpropanoid catabolism genes strongly suggests the physiological role of the enzyme is to release C1 moieties from PPMEs. Accordingly, we refer to PFLU3311 as *pme.*

### PFLU3296 encodes a phloretyl-CoA dehydrogenase

Disruption of the remaining two genes, PFLU3296 and PFLU3297, incurs a large negative fitness impact when using MHPP and phloretate as the growth substrate, but not methyl *p*-coumarate (Fig. 3—see cyan dots). These two genes are the last two genes in the *P. fluorescens ech* operon (PFLU3296 and PFLU3297) and encode putative acyl-CoA dehydrogenase and a β-ketothiolase, respectively. The predicted enzymatic functions and fitness data suggest a role in oxidation of the saturated propyl moieties of MHPP and phloretate. To test the proposed functions, we generated targeted deletions of each gene and evaluated the ability of the resulting mutants to use each growth substrate (Fig. 4B). Deletion of PFLU3296 abolished growth with MHPP and phloretate. However, deletion of PFLU3297 had limited impact on growth. To confirm that the loss of growth with MHPP and phloretate was not due to a polar effect from the gene deletion, we integrated a constitutively expressed copy of PLFU3296 at a separate site in the chromosome and evaluated the ability of the resulting strain to utilize MHPP. Consistent with the proposed function, ectopic expression of PFLU3296 complemented the deletion by allowing the strain to grow using MHPP (Supplementary Fig. S6). We propose to name PFLU3296 gene *pcd* for phloretoyl-CoA dehydrogenase.

### Disruption of putative aromatic transporters has a minor impact on aromatic catabolism

Transposon mutants for two putative transporters exhibited variable fitness scores across phenylpropanoid growth substrates. For PFLU3302, which encodes a putative porin, we observed high positive fitness values when using ferulate or *p-*coumarate as carbon sources. When using other phenylpropanoids the fitness was either not affected (methyl *p*-coumarate), neutral, or mildly negative (MHPP). Although the high fitness value indicates that the PFLU3302 mutant had a strong growth advantage over other mutant strains in a community, the effect of gene deletion in axenic culture was mild with only a minor improvement in growth with p-coumarate and phloretate (Supplementary Fig. S8). We also observed moderate negative fitness values for PFLU3303, a putative phenylpropanoid transporter, during growth with MHPP. However, targeted deletion had little impact on growth with any of the tested phenylpropanoids (Supplementary Fig. S8). Together these data suggest that there is redundancy in phenylpropanoid transport.

### The mechanism for catabolism of the C1 moiety is unclear

A C1 moiety, presumably methanol, is expected to be released by *pme*, but it is unclear in these data how and if this compound is metabolized in *P. fluorescens*. The related bacteria, *P. putida* KT2440, contains two periplasmic pqq-dependent alcohol dehydrogenases that are closely related to the methanol dehydrogenases found in methylotrophic bacteria. These enzymes are responsible for oxidation of short chain *n*-alcohols by *P. putida* [63, 64]. However, there are no clear orthologs of these proteins encoded in the *P. fluorescens* genome, and it seems unlikely that periplasmic alcohol dehydrogenases would function on intracellular C1 compounds. Surprisingly, we did not observe other differentially expressed putative alcohol, aldehyde, or formate dehydrogenases during growth with MHPP (Supplementary File F2). Similarly, there was not a clear connection between fitness values for genes that encode these classes of enzymes and PPME compounds (Supplementary File F1). As such, it remains unclear exactly how or if the C1 moiety released during PPME catabolism is metabolized by *P. fluorescens*. Without dedicated enzymes the slow mineralization of methanol to CO_2_ via a toxic formaldehyde intermediate (see Fig. 4) may explain the apparent toxicity associated with Pme expression in the presence of MHPP (positive fitness score in Fig. 3A).

### A proposed pathway for phenylpropanoid methyl ester catabolism

**Figure 6:**
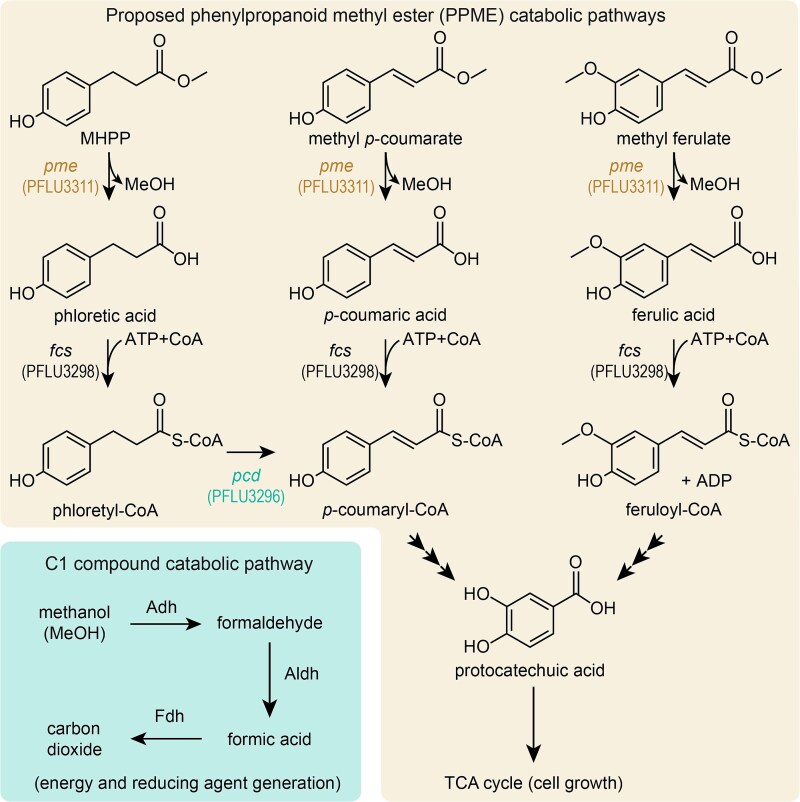
A proposed pathway for PPME catabolism. Proposed pathways for catabolism of several different PPMEs. Pathways in the top and bottom right follow catabolism of the phenylpropanoid moiety following an initial de-esterification step. The pathway in the bottom left is a standard C1 metabolic pathway that follows the MeOH moeity released upon de-esterification. Adh (alcohol dehydrogenase), aldehyde dehydrogenase (Aldh), and formate dehydrogenase (Fhd) enzymes are indicated in the proposed C1 pathway.

Through integrated analysis of differential transcriptomic and mutant fitness measurements, we propose a pathway for PPME catabolism (Fig. 6). First, phenylpropanoid methyl esterase, encoded by *pme* (PFLU3311), hydrolyzes the PPMEs, releasing an aromatic acid and methanol. The aromatic acids are then ligated to CoA by feruloyl-CoA synthetase and metabolized through parallel pathways (Fig. 6—tan fill). In our proposed pathway phloretoyl-CoA, the product of CoA ligation to phloretate, is subsequently oxidized by phloretoyl-CoA dehydrogenase, which is the product of *pcd* (PFLU3296), into *p-*coumaroyl-CoA which is a metabolic intermediate of the known *p-*coumarate catabolic pathway.

To add evidence for our proposed pathway, we engineered multiple *Pseudomonas* species to use PPMEs as carbon sources. *Pseudomonas putida* KT2440, *P.* sp TBS28, and *P. frederiksbergensis* TBS10 each contain a subset of the genes we identified as contributing to PPME catabolism (Supplementary Fig. S4); we predicted that introducing PFLU3311 and PFLU3296 into these organisms would allow growth on PPME. We transferred constitutively expressed copies of *pme*, *pcd*, or both genes, into the chromosomes of *P. putida*, *P.* sp TBS28 and *P. frederiksbergensis*, respectively (Supplementary Fig. S9). *P.* sp TBS28 encodes an ortholog of *pme* near its *ech* operon but lacks *pcd*. *Pseudomonas putida* contains an ortholog of *pcd* in its *ech* operon but lacks *pme*. We found that expression of these genes enabled each strain to use PPMEs as carbon sources and that expressing a fluorescent protein as a negative control did not confer growth (Supplementary Fig. S9). However, *P. frederiksbergensis*, and to a lesser extent *P.* sp. TBS28, catabolized MHPP slowly when expressing these genes. Taken together, these findings solidify evidence that the enzymes encoded by *pme* and *pcd* are critical for catabolism of PPMEs and demonstrates a relatively low barrier for horizontal transfer of PPME catabolic capability. Although the strains are able to utilize MHPP, growth was relatively slow. The slow growth may be due to inefficient transport. This may be a consequence of poor expression or low substrate affinity between MHPP and native transport proteins. Alternatively, slow growth may be indicative of poor activation of downstream pathways by MHPP (e.g. poor expression of *fcs*).

## Discussion

Enhancing BNI has been proposed as a strategy to mitigate agricultural nitrogen pollution [14]. However, our understanding of how BNI compounds are broken down within microbial ecosystems—and to what extent—is currently very limited. This knowledge gap presents a major hurdle in designing effective and targeted interventions. Predicting the success of BNI applications is particularly challenging because we lack reliable tools to quickly assess the microbial community’s capacity to degrade specific BNIs. A potential breakthrough is to identify genes that encode enzymes responsible for breaking down BNIs and using them as functional molecular markers. These molecular markers could be analyzed with current molecular methods, such as 16S rRNA gene sequencing or other PCR assays. Although developing these approaches is outside of the scope of this study, using the presence and expression of genes encoding BNI degrading enzymes in a microbial community as a molecular signature could provide new insights into ecological functions.

In this study, we identified a catabolic pathway in *P. fluorescens* SBW25 that encodes enzymes enabling the breakdown of a class of aromatic BNI compounds. To achieve this, we developed a pathway discovery approach that combines differential transcriptomics with genome-wide mutant fitness screens. These two complementary methods for identifying genes in metabolic pathways [40, 65–69] are synergistic, and their combined usage enabled PPME pathway discovery by narrowing the pool of candidate genes to an experimentally manageable number. Catabolic pathways for aromatic compounds often converge into small group of established pathways [36, 70], such as the phenylpropanoid catabolic pathway used for *p-*coumarate. Given its structure, we hypothesized that MHPP (a PPME-derived intermediate) would similarly be processed through one of these pathways. Using omics, genetic, and biochemical analyses, we identified two key enzymes—putative serine hydrolase (Pme, encoded by *PFLU3311*) and acyl-CoA dehydrogenase (Pcd, encoded by *PFLU3296*)—that catalyze these processes. Pme is an enzyme in a previously unknown family of methyl esterases, serving as the pivotal enzyme for PPME catabolism. Although we elucidated the pathway for the aromatic component of PPMEs, our data do not point to a specific pathway for metabolizing the methanol released during PPME de-esterification. Furthermore, this study assigns new functions to previously uncharacterized genes, including the *pcd* ortholog in *P. putida* and the *pme* ortholog in *Pseudomonas sp.* TBS28.

*Pseudomonas fluorescens* SBW25—originally isolated from sugar beets—was found to metabolize a BNI compound currently known to be exuded only by sorghum roots [15]. However, none of the sorghum endosphere isolates we tested were able to metabolize MHPP as a carbon source. This difference may reflect variations in their environmental niches. It was previously found that *P. fluorescens* isolates from the rhizosphere and endosphere of the same poplar plants are enriched for distinct sets of metabolic pathways [71]. Specifically, pathways for root exudate compounds were substantially less prevalent in endophytes, such as *Pseudomonas sp.* TBS28 and *P. frederiksbergensis* TBS10, compared to isolates from the plant’s rhizosphere. These findings suggest that PPME exudation by plant roots might be more widespread than currently documented, as demonstrated by SBW25’s ability to metabolize them despite originating from a different plant species.

## Supplementary Material

PPME_supplemental_-_final_wraf251

Supplemental_File_F1_wraf251

Supplemental_File_F2_wraf251

Supplemental_File_F3_wraf251

Supplementary_File_P1_wraf251

## Data Availability

Datasets generated during and/or analysed during the current study are available in the Gene Expression Omnibus (GEO) repository, https://www.ncbi.nlm.nih.gov/geo/query/acc.cgi?acc=GSE228022.
