# Supplementary material for: Phenylpropanoid methyl esterase unlocks catabolism of aromatic biological nitrification inhibitors
Source: ISME J. 2025 Nov 13;19(1):wraf251. doi: 10.1093/ismejo/wraf251 (PMC12642758; doi:10.1093/ismejo/wraf251)
Supplement: PPME_supplemental_-_final_wraf251 [file ppme_supplemental_-_final_wraf251.docx]

**­Phenylpropanoid methyl esterase unlocks catabolism of aromatic biological nitrification inhibitors**

**Supplementary Material**


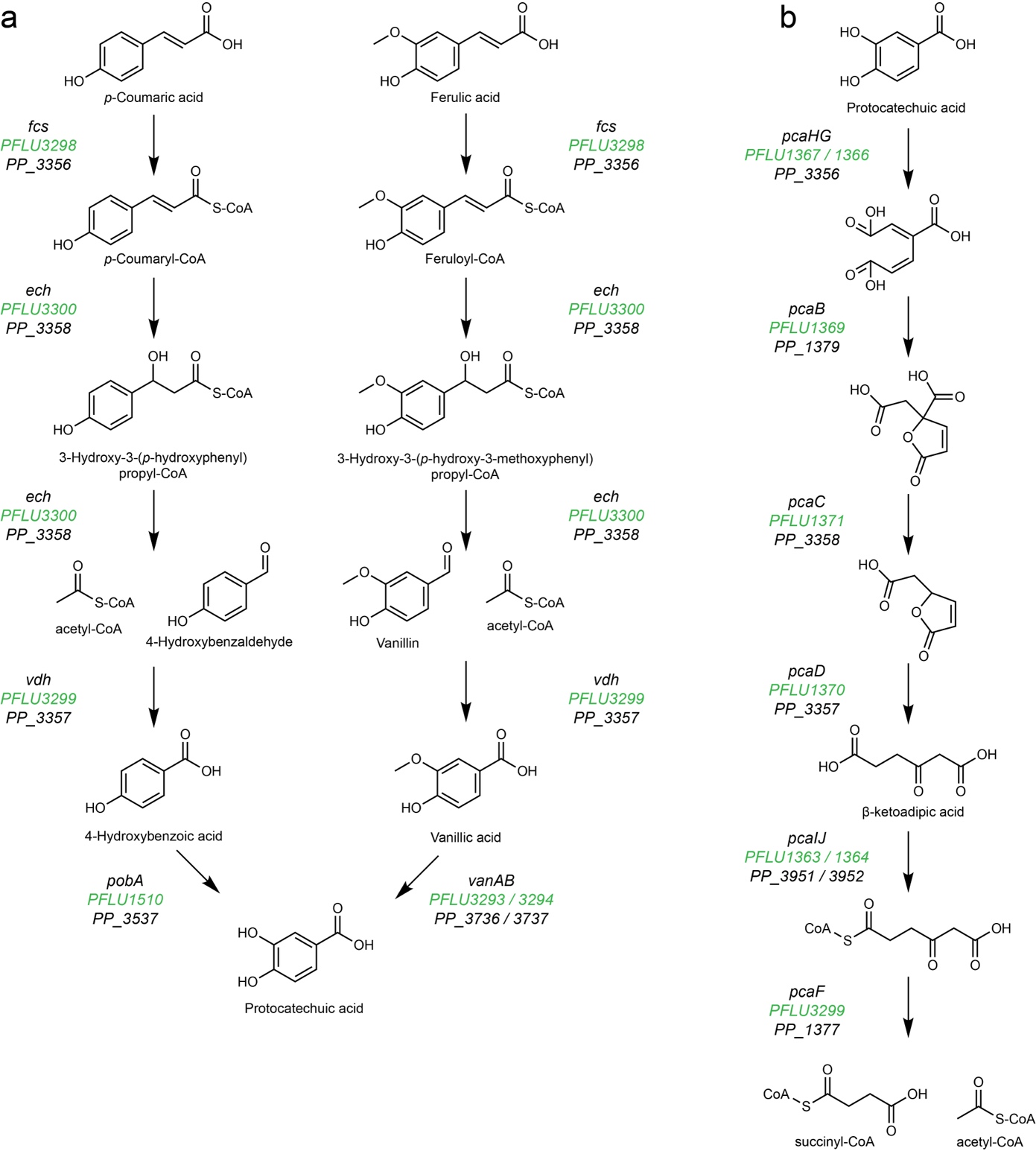


**Supplementary Fig. S1. Catabolic pathways for *p­*-coumaric and ferulic acids.** (a) Convergent portion of the catabolic pathways for the two phenylpropanoid carbon sources, which is largely comprised of enzymes encoded in the *ech* gene cluster. (b) Ortho-cleavage pathway for protocatechuic acid, which is also commonly referred to as the B-ketoadipate pathway after its characteristic intermediate. This portion of the pathway funnels the aromatic compounds into the TCA cycle. Names of the genes encoding the enzymes that perform each step are shown in *italics* and locus tags for *P. fluorescens* SBW25 and *P. putida* KT2440 are shown in green and black text, respectively.

**Supplementary Fig. S2. A subset of environmental Pseudomonas isolates can utilize phenylpropanoid compound phloretic acid as a sole carbon source.** Microtiter plate cultivation assays comparing growth of four environmental Pseudomonads in MME medium containing 2.5 mM phloretic acid as the sole carbon source. Panel contains a single representative curve from one of three biological replicates.


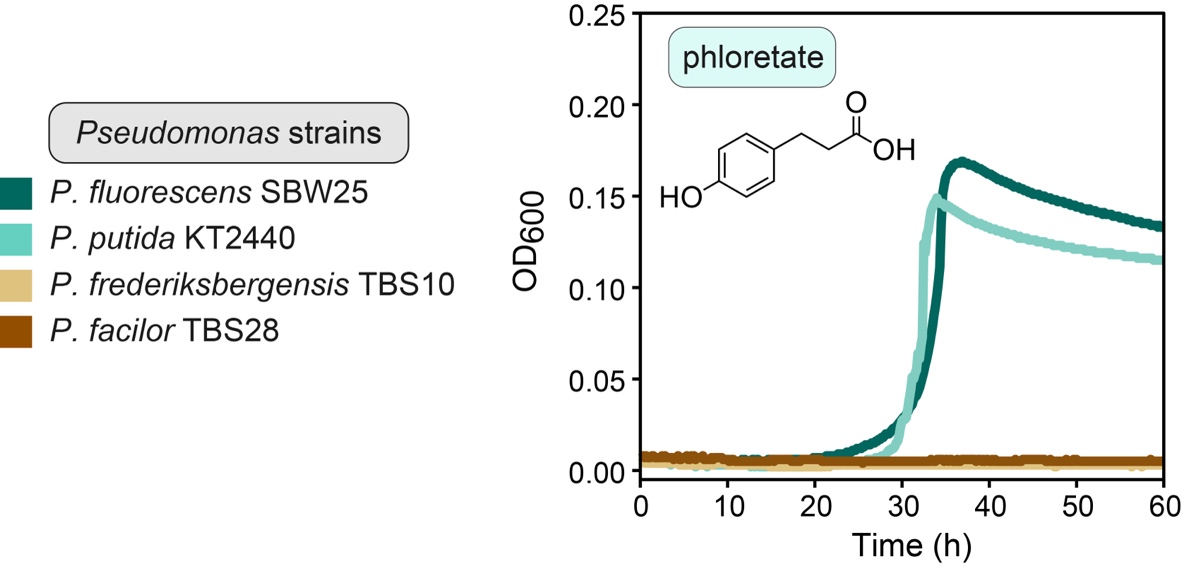


**Supplementary Fig. S3. Chart of differential gene expression when *P. fluorescens* SBW25 was grown with either glucose or MHPP as the sole carbon source.** Positive values on the X-axis indicate genes whose expression was higher when MHPP was the carbon source than when glucose was the carbon source. The y-axis represents a log transformation of the p-value that has been adjust for multiple tests. Brown and teal colored dots indicate genes whose differential expression was statistically significant and greater than 8-fold in crease in expression during growth on MHPP (brown) or glucose (teal). Samples with an adjusted p-value of 0 are shown at the top of the Y-axis as triangles, as they cannot be plotted otherwise.


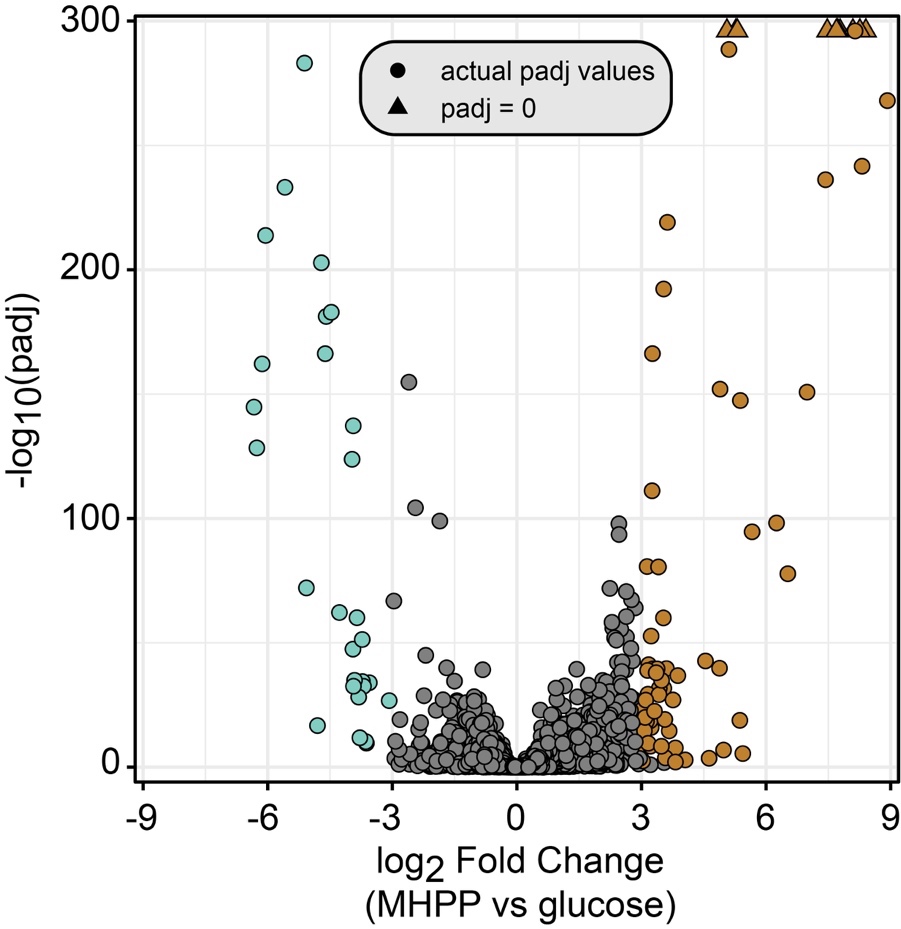

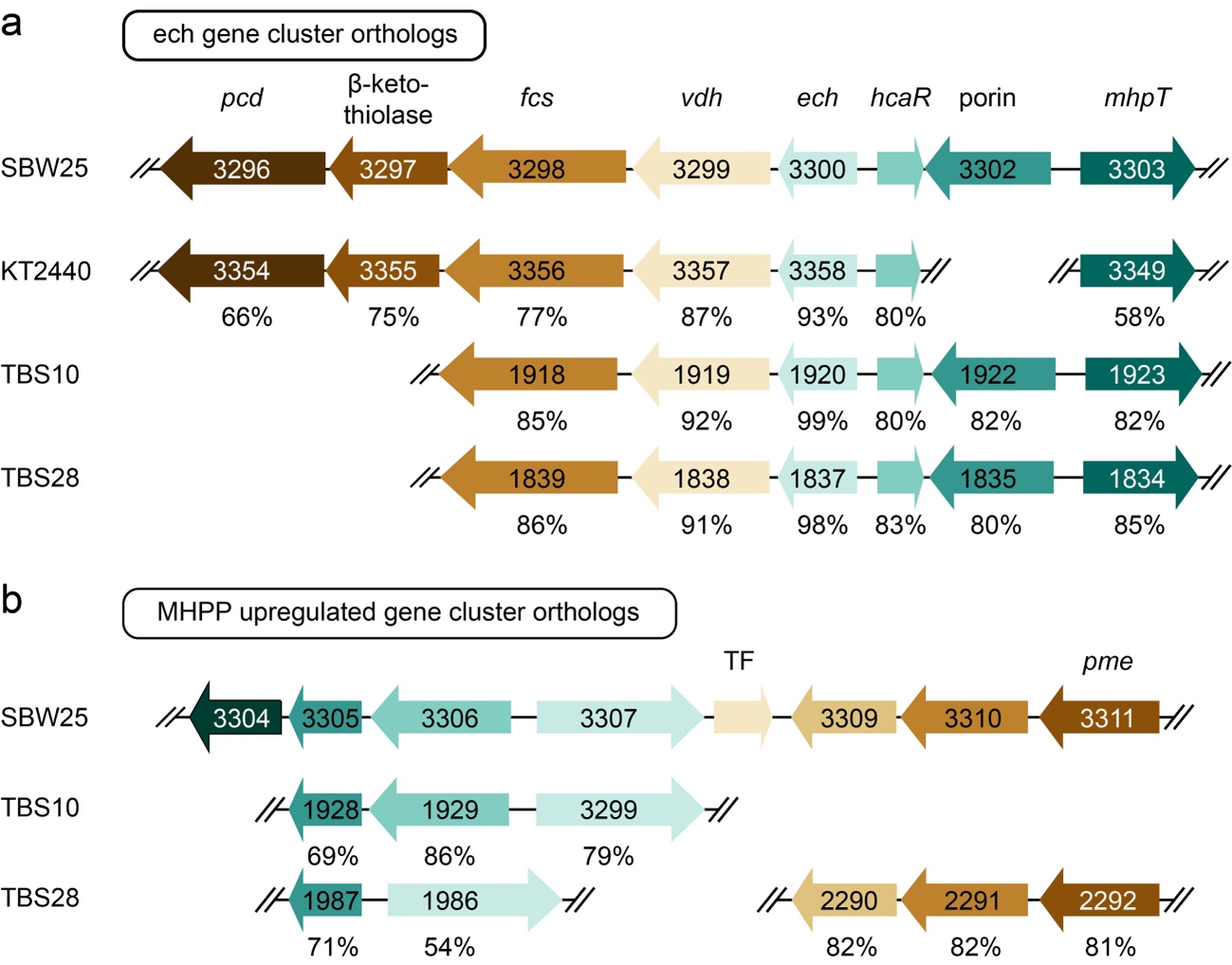


**Supplementary Fig. S4. Alignments of differentially expressed gene clusters containing genes critical for growth on MHPP and other phenylpropanoids in SBW25 with orthologs from other environmental Pseudomonads.** Locus tag numbers for genes in each organism are indicated in the arrows, with the exception of locus tags for *hcaR* and PFLU3308. Gene arrangement and spacing is maintained in each organism. Coloring in the arrows indicates orthologous genes. Percentage values under each arrow indicate the % identity of the SBW25 gene with the closest matching genes in KT2440, TBS10, or TBS28 gene. Genes with less than 50% identity were not considered. In organisms other than SBW25 genes from the *ech* gene cluster (a) are not co-localized with their equivalents of the MHPP upregulated gene cluster (b).

**Supplementary Fig. S5. Comparing fitness values with differential gene expression reduces search space for novel pathway genes.** (a-d) Plots comparing differential expression (x-axis) versus mean RB-TnSeq fitness values on the y-axis. RB-TnSeq values displayed are from cultures grown with either (a) *p*-coumarate, (b) ferulate, (c) 4-hydroxybenzoate (a downstream metabolite of *p*-coumaric acid catabolism), or (d) glucose. Positive and negative differential expression values indicate higher expression during growth using MHPP and glucose as carbon sources, respectively. Dots indicate genes encoding the putative PPME-sensitive *pmeR* transcription factor (dark teal), phenylpropanoid methyl esterase (light brown), putative phloretoyl-CoA dehydrogenase and putative β-ketothiolase (light teal), other genes in the *ech* gene cluster (dark brown), other genes in the MHPP-upregulated gene cluster (medium brown), downstream aromatic catabolic pathway gene clusters (white), and all other genes (dark gray). Values represent the mean of 4 RB-TnSeq or 4 differential expression biological replicates. Genes lacking fitness or differential expression values are not displayed.


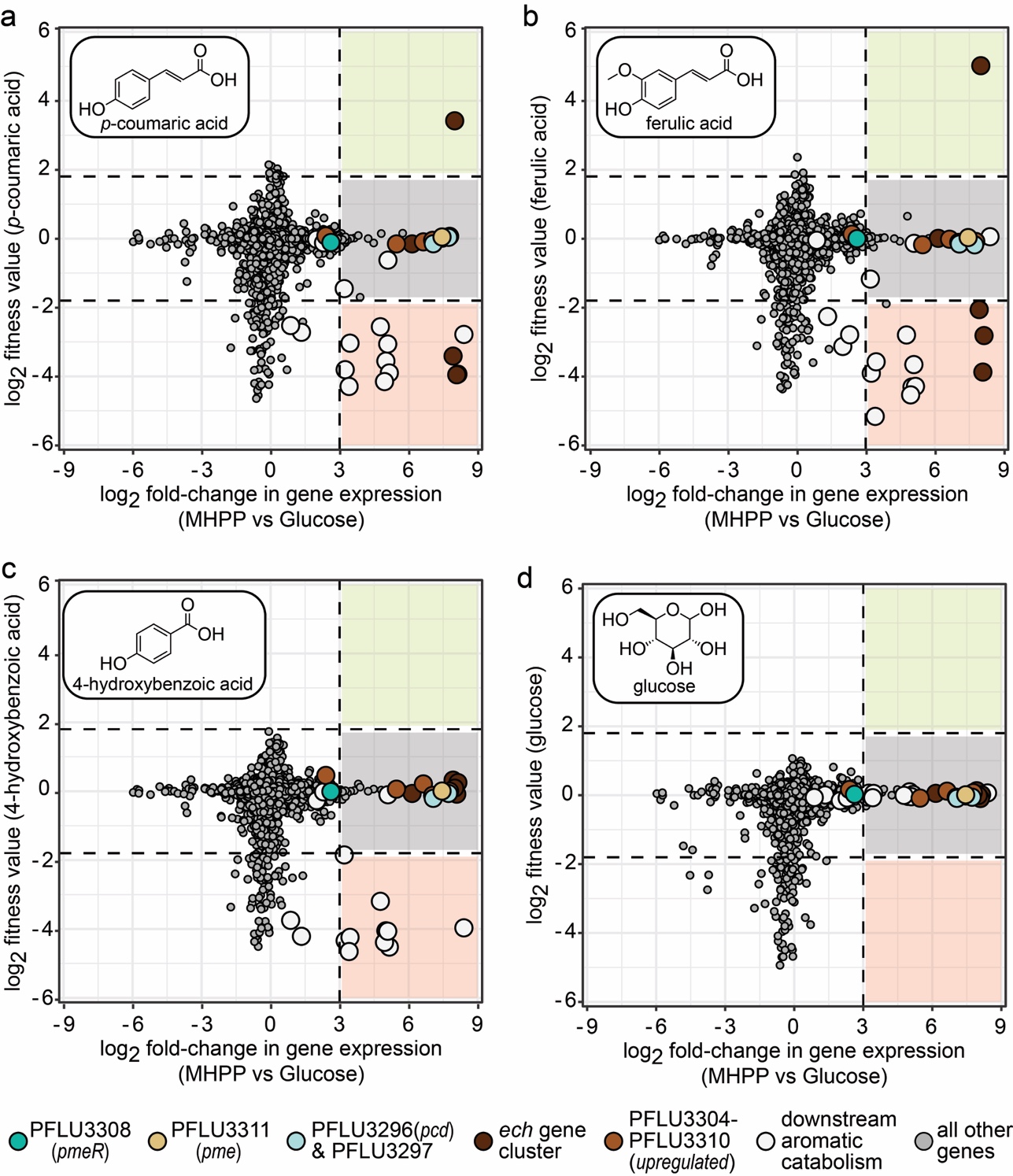


**Supplementary Fig. S6. Complementation of PFLU3296 and PFLU3311 gene deletions enables growth with MHPP and phloretic acid carbon sources.** Microtiter plate cultivation assays comparing growth of five *P. fluorescens* strains in MME medium containing 2.5 mM *p*-coumarate or 2.5 mM MHPP as the sole carbon source. Each panel contains a single representative curve from one of three biological replicates.

**
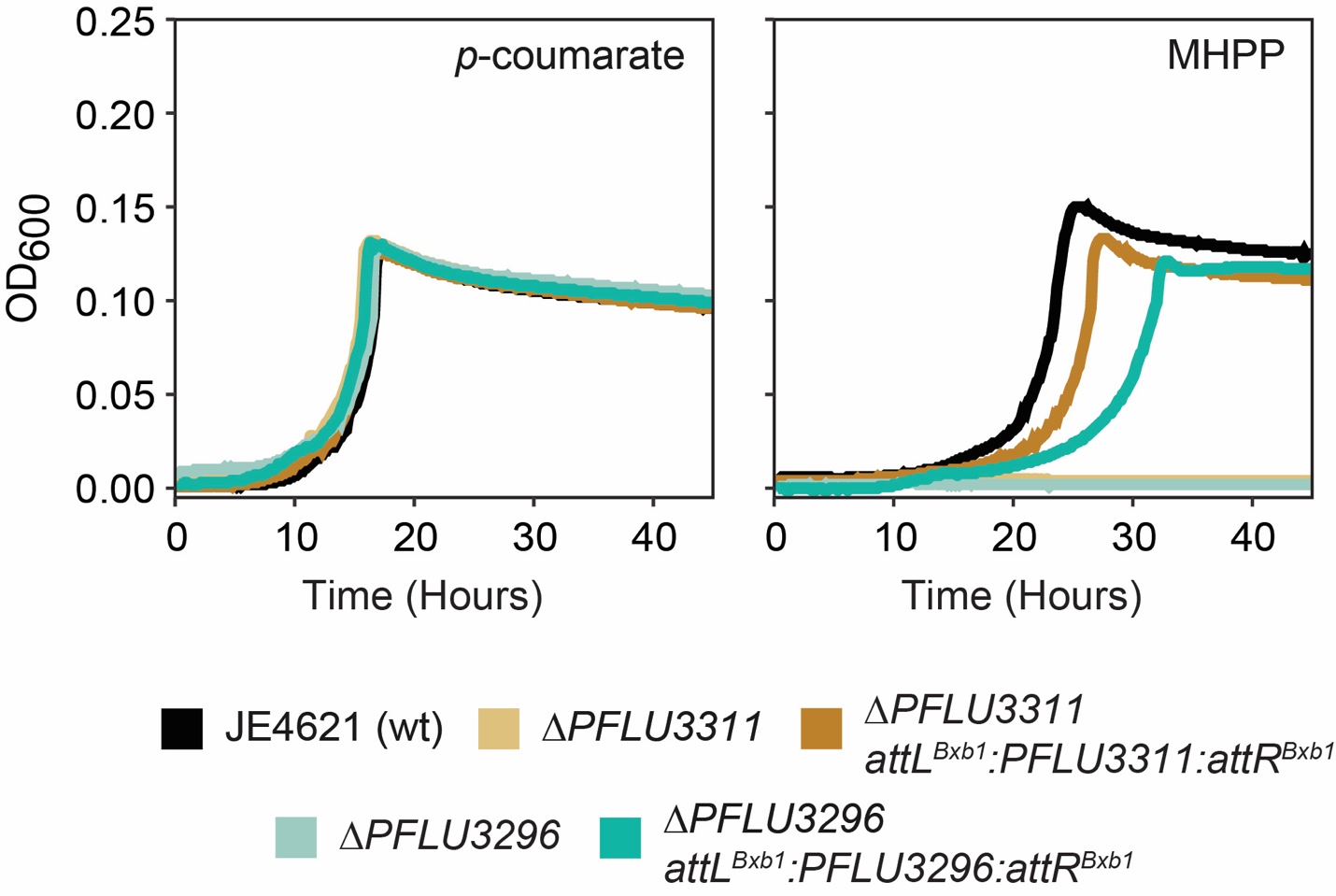

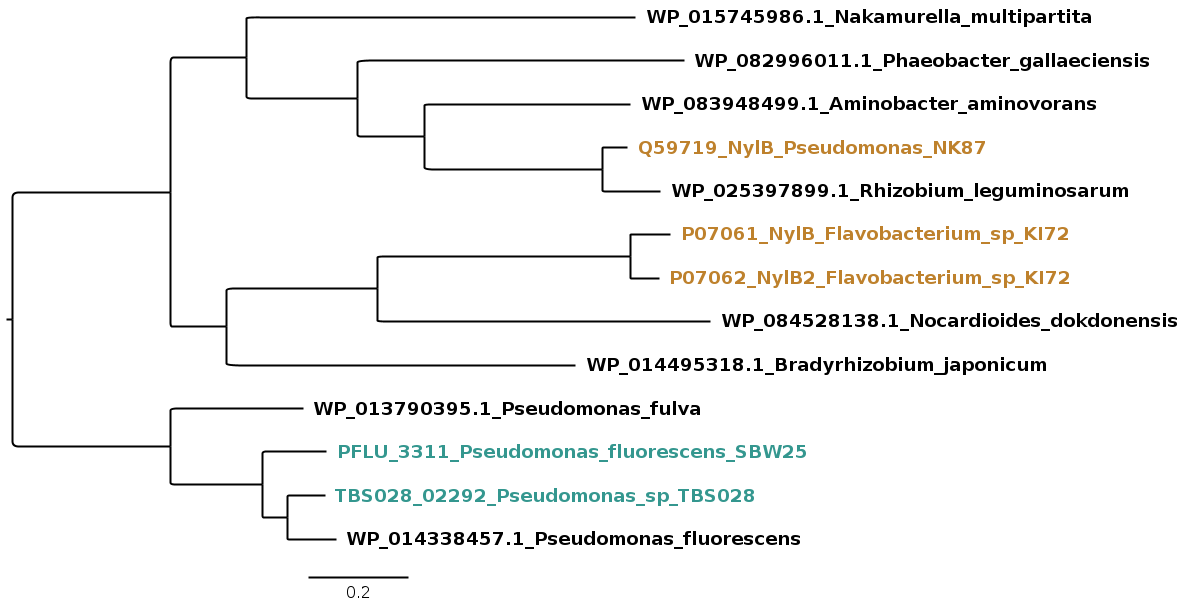
**

**Supplementary Fig. S7. Phylogenetic tree of serine hydrolases.** Serine hydrolase sequences from P. fluorescens SBW25 (teal) and Pseudomonas sp. TBS028 (teal) were aligned against proteins with known 6-aminohexanoate-dimer hydrolase activity (tan) and a selection of uncharacterized proteins with the closest sequence similarity to the methyl esterases (black). The proteins of interest only share ~35% AAID with the KI72 NylB and NylB2 sequences [1, 2].

**Supplemental Fig. S8. Deletion of putative aromatic compound transporters have varying impacts upon growth when using phenylpropanoids as carbon sources.** Microtiter plate cultivation assays comparing growth of three *P. fluorescens* strains in MME medium containing 2.5 mM *p*-coumaric acid, 2.5 mM MHPP, or 2.5 mM phloretic acid as the sole carbon source. Each panel contains a single representative curve from one of three biological replicates.


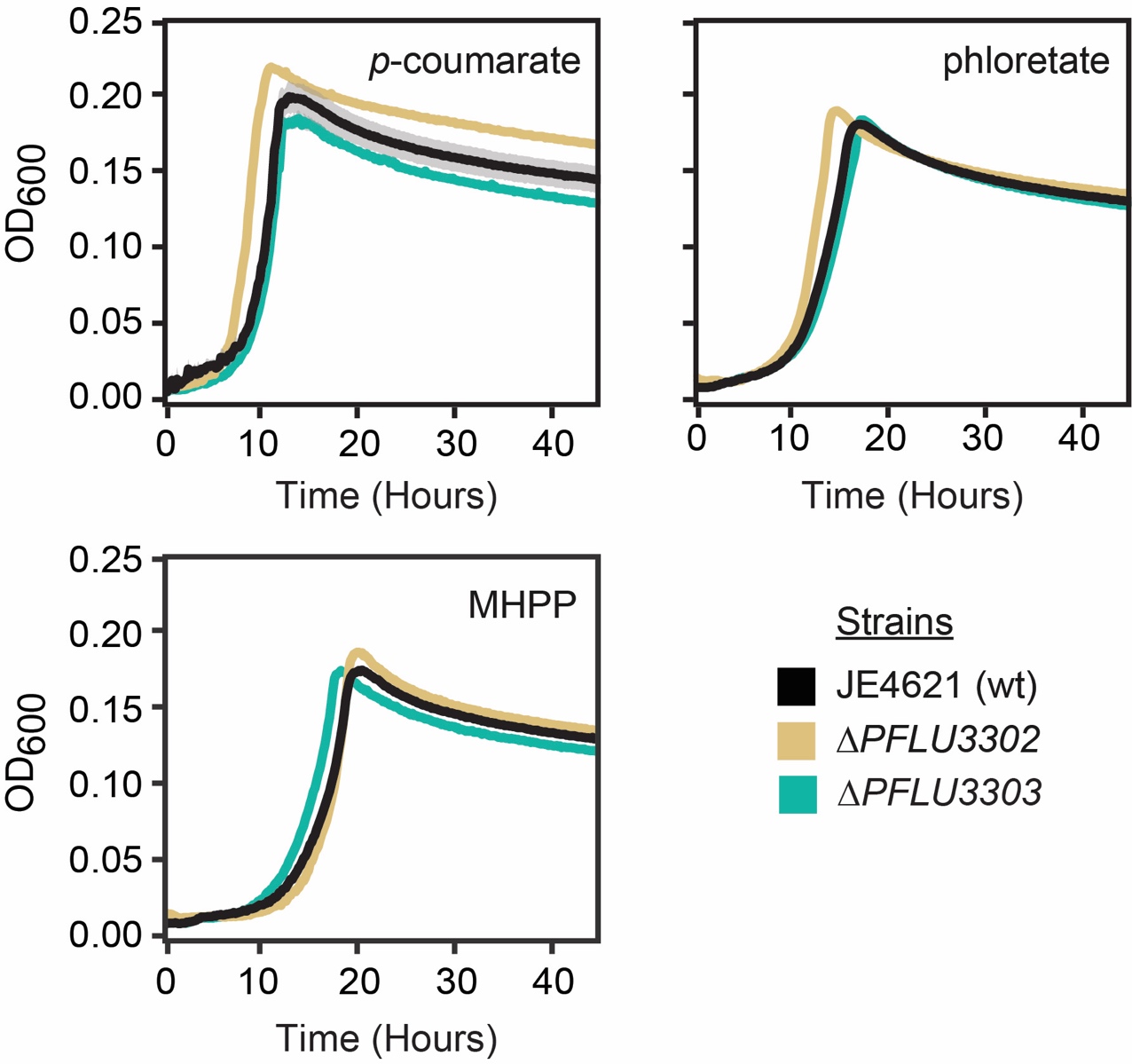

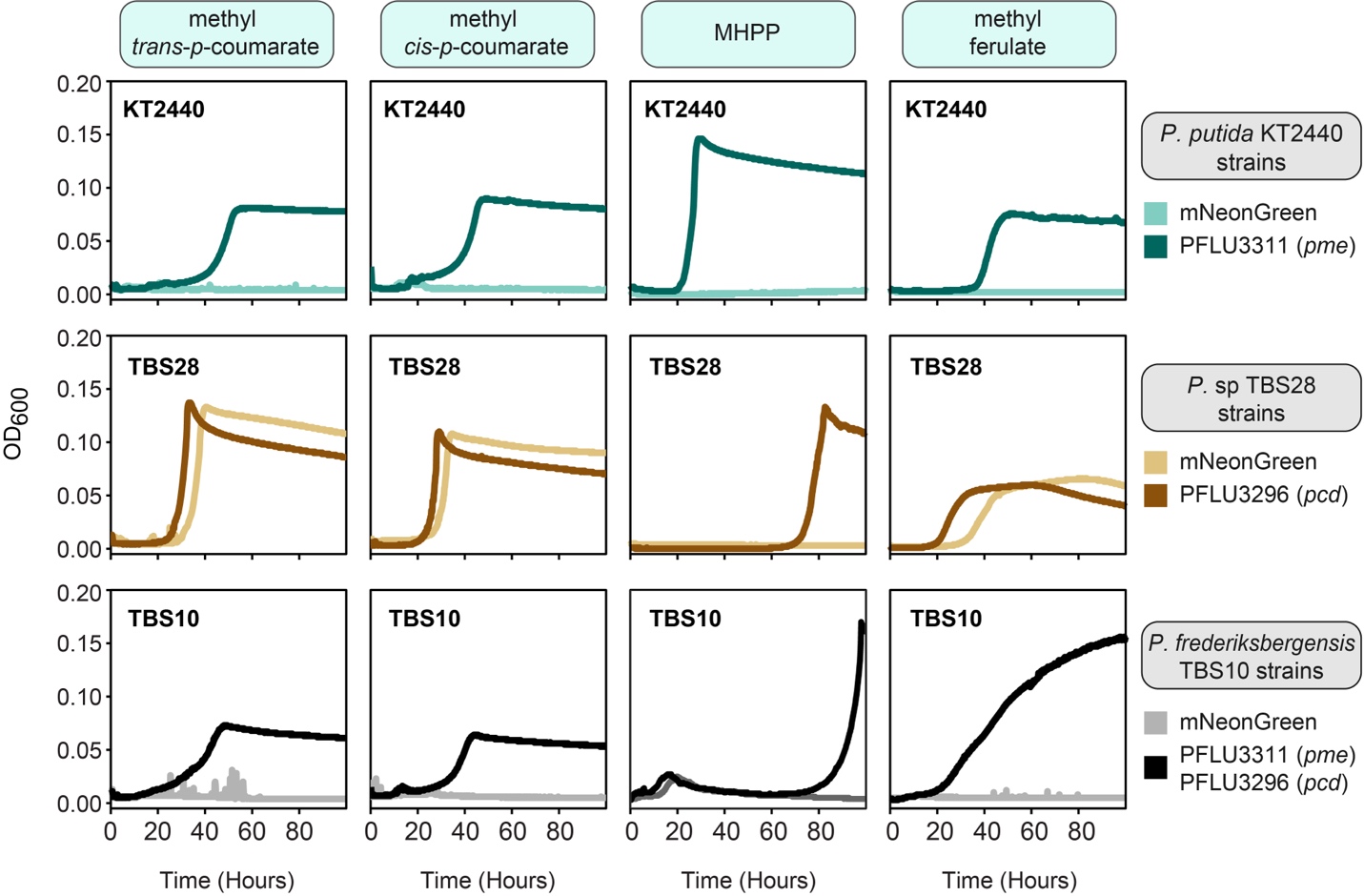


**Supplementary Fig. S9. Transfer of *pme* and *pcd* genes enables heterologous hosts to use PPMEs as carbon sources.** Microtiter plate cultivation assays comparing growth of Pseudomonads expressing either the fluorescent protein mNeonGreen or the Pme/Pcd enzymes from SBW25. Assays were performed with MME medium containing 2.5 mM of the indicated carbon source. Each panel contains a single representative curve from one of three biological replicates.

| **Supplementary Table S1. Strains and Plasmids used in this study.** | |  |
| --- | --- | --- |
| **Name** | **Relevant Genotype** | **Source** |
| *Strains* |  |  |
| NEB 5-alpha F'Iq | *Escherichia coli* F´ *proA^+^B^+^ lacI^q^ ∆(lacZ)M15 zzf::Tn10* (Tet^R^) */ fhuA2∆(argF-lacZ)U169 phoA glnV44 Φ80Δ(lacZ)M15 gyrA96 recA1 relA1 endA1 thi-1 hsdR17* | New England Biolabs |
| SBW25 | *Pseudomonas fluorescens SBW25* | [3] |
| KT2440 | *Pseudomonas putida* KT2440 | [4] |
| TBS10 | *Pseudomonas frederiksbergensis* TBS10 | [5] |
| TBS28 | Pseudomonas sp. TBS28 | this work |
| Pf-5 | *Pseudomonas protegens* Pf-5 | [6] |
| DSM4188 | *Pseudomonas stutzeri* DSM4166 | [7] |
| TBS49 | *Pseudomonas* sp. TBS49 | this work |
| JE4621 | *P. fluorescens* SBW25 3’ *ampC*:poly-*attB* | [5] |
| JE90 | *P. putida* KT2440 ∆hsdR::Bxb1int-*attB* | [8] |
| RS175 | *P. facilor* 3’ *ampC*:poly-*attB* | this work |
| JE5041 | *P. frederiksbergensis* TBS10 *att*^Tn5^:10x poly-*attB*:*att*^Tn5^ | this work |
| RS137 | *P. fluorescens* JE4621 ∆PFLU3296 (pcd) | this work |
| AW65 | *P. fluorescens* JE4621 ∆PFLU3297 | this work |
| AW54 | *P. fluorescens* JE4621 ∆PFLU3298 (*fcs*) | this work |
| AW51 | *P. fluorescens* JE4621 ∆PFLU3300 (*ech*) | this work |
| RS183 | *P. fluorescens* JE4621 ∆PFLU3302 | this work |
| RS184 | *P. fluorescens* JE4621 ∆PFLU3303 | this work |
| AF001 | *P. fluorescens* JE4621 ∆PFLU3311 (*pme*) | this work |
| AW70 | *P. fluorescens* JE4621 ∆PFLU3296 *attL*^Bxb1^:pAW30:*attR*^Bxb1^ | this work |
| AW71 | *P. fluorescens* JE4621 ∆PFLU3311 *attL*^Bxb1^:pJE1918:*attR*^Bxb1^ | this work |
| AW72 | *P. fluorescens* JE4621 ∆PFLU3311 *attL*^Bxb1^:pJE1944:*attR*^Bxb1^ | this work |
| AW74 | *P. fredericksbergensis* JE5041 *attL*^Bxb1^:pJE1920:*attR*^Bxb1^ | this work |
| AW73 | *P. putida* JE90 *attL*^Bxb1^:pJE1918:*attR*^Bxb1^ | this work |
| AW75 | *P*. sp RS175 *attL*^Bxb1^:pAW30:*attR*^Bxb1^ | this work |
| AW76 | *P. fredericksbergensis* JE5041 *attL*^Bxb1^:pJE1045:*attR*^Bxb1^ | this work |
| AW77 | *P. putida* JE90 *attL*^Bxb1^:pJE1045:*attR*^Bxb1^ | this work |
| AW78 | *P*. sp. RS175 *attL*^Bxb1^:pJE1045:*attR*^Bxb1^ | this work |
|  |  |  |
| *Plasmids* |  |  |
| pK18sB | pUC origin, *nptII*, *sacB* | [9] |
| pGW31 | pUC origin, AprR*,* P_tac_:*Bxb1 integrase* ∆mSF^ts1^ | [5] |
| pJE1045 | pJE990 P_tac_:*mNeonGreen* | [10] |
| pGW60 | pJE990 P_tac-mod_:*mNeonGreen,* 10x poly-*attP* cassette | [5] |
| pJE1918 | pJE990 P_tac_:PFLU3311 | this work |
| pJE1920 | pJE990 P_tac_:PFLU3311:PFLU3296 | this work |
| pAW30 | pJE990 P_tac_:PFLU3296 | this work |
| pRS329 | pK18sB 3’*ampC*:poly-*attB* in TBS28 | this work |
| pRS309 | pK18sB ∆PFLU3296 | this work |
| pAW5 | pK18sB ∆PFLU3297 | this work |
| pAW11 | pK18sB ∆PFLU3298 | this work |
| pAW6 | pK18sB ∆PFLU3300 | this work |
| pRS337 | pK18sB ∆PFLU3302 | this work |
| pRS338 | pK18sB ∆PFLU3303 | this work |
| pAF002 | pK18sB ∆PFLU3311 | this work |
| pEVF-SBP1-P2 | PFLU3311 expression vector | this work |

**Supplementary Table S2. Oligos**

| Name | | Sequence | | Description | |
| --- | --- | --- | --- | --- | --- |
| oPNL617-pK18sB_scr_F | | GAGCGTCGATTTTTGTGATG | | Forward screening primer for pK18sB constructs | |
| oPNL618-pK18sB_scr_R | | TTGCAGGGCTTCCCAACCT | | Reverse screening primer for pK18sB constructs | |
| oPNL1471 (PFLU3296_UP arm _F) | | aacagctatgacatgattacgaattcctatcagcccagagggatc | | Forward primer for PFLU_3296 upstream homology arm | |
| oPNL1472 (PFLU3296_UP arm _R) | | gtcagtaccgcaagcacctggccgcgcggcacacctcatcgacta | | Reverse primer for PFLU_3296 upstream homology arm | |
| oPNL1473 (PFLU3296_DN arm _F) | | gattcctttttagtcgatgaggtgtgccgcgcggccaggtgcttgcgg | | Forward primer for PFLU_3296 downstream homology arm | |
| oPNL1474 (PFLU3296_DN arm _R) | | gtaaaacgacggccagtgccaagcttcatagttgaagcgtcggtat | | Reverse primer for PFLU_3296 downstream homology arm | |
| oPNL1475 (PFLU3296_scrF) | | cgtgacggcgcctgggctg | | Forward flanking primer for JE4621∆PFLU_3296 | |
| oPNL1476 (PFLU3296_scrR) | | agggcatcgaagaccgagtg | | Reverse flanking primer for JE4621∆PFLU_3296 | |
| oPNL1477 (PFLU3296_intF) | | gcgcgatatgcagtttgtg | | Forward internal primer for JE4621∆PFLU_3296 | |
| oPNL1478 (PFLU3296_intR) | | gaacgccagcgctgcccg | | Reverse internal primer for JE4621∆PFLU_3296 | |
| PFLU3297_up_F | | aacagctatgacatgattacgaattccatcatcacaaacatcgccgccag | | Forward primer for PFLU_3297 upstream homology arm | |
| PFLU3297_up_R | | acccaagccttgagcggaccctgcctatcgcgcttgaa | | Reverse primer for PFLU_3297 upstream homology arm | |
| PFLU3297_down_F | | caagcgcgataggcagggtccgctcaaggcttgggtcc | | Forward primer for PFLU_3297 downstream homology arm | |
| PFLU3297_down_R | | tgtaaaacgacggccagtgccaagcttgagaccctgcgcaacctcaa | | Reverse primer for PFLU_3297 downstream homology arm | |
| PFLU_3297_screen_F | | atggccgggtggtaacgga | | Forward flanking primer for JE4621∆PFLU_3297 | |
| PFLU3297_screen_R2 | | ttcggtgaggaaccgccggtactggt | | Reverse flanking primer for JE4621∆PFLU_3297 | |
| PFLU_3297_int_F | | atcccgtagcgcaggtggtg | | Forward internal primer for JE4621∆PFLU_3297 | |
| PFLU_3297_int_R | | atatcggcttgtacagcggc | | Reverse internal primer for JE4621∆PFLU_3297 | |
| PFLU3298_up_F | | aacagctatgacatgattacgaattcgttaccggcggtttgtacacc | | Forward primer for PFLU_3298 upstream homology arm | |
| PFLU3298_up_R | | gccgctgggtgaattaagacccgaccatcctgcgc | | Reverse primer for PFLU_3298 upstream homology arm | |
| PFLU3298_down_F | | ggatggtcgggtcttaattcacccagcggctccag | | Forward primer for PFLU_3298 downstream homology arm | |
| PFLU3298_down_R | | tgtaaaacgacggccagtgccaagcttatgcgttcaccgccaagct | | Reverse primer for PFLU_3298 downstream homology arm | |
| PFLU3298_scr_F | | aattgccggtgccggacc | | Forward flanking primer for JE4621∆PFLU_3298 | |
| PFLU3298_scr_R | | cggcagcatttggtgcctact | | Reverse flanking primer for JE4621∆PFLU_3298 | |
| PFLU3298_int_F | | acgccggctcatccaacaca | | Forward internal primer for JE4621∆PFLU_3298 | |
| PFLU3298_int_R | | ctgagttgcgcgagcgctt | | Reverse internal primer for JE4621∆PFLU_3298 | |
| PFLU_3300_up_F | | aacagctatgacatgattacgaattccagcttggcggtgaacgcat | | Forward primer for PFLU_3300 upstream homology arm | |
| PFLU_3300_up_R | | gaacaagagcgtaccgtcatggcctgcaggcgtataaacg | | Reverse primer for PFLU_3300 upstream homology arm | |
| PFLU_3300_down_F | | ttatacgcctgcaggccatgacggtacgctcttgttctg | | Forward primer for PFLU_3300 downstream homology arm | |
| PFLU_3300_down_R | | tgtaaaacgacggccagtgccaagcttactacgatttcgccgcagc | | Reverse primer for PFLU_3300 downstream homology arm | |
| PFLU_3300_screen_F | | cagcgaccccagcactgaat | | Forward flanking primer for JE4621∆PFLU_3300 | |
| PFLU_3300_screen_R | | ccaaggagcgcacgtggca | | Reverse flanking primer for JE4621∆PFLU_3300 | |
| PFLU_3300_int_F | | aggcttgatgctcttgtcgt | | Forward internal primer for JE4621∆PFLU_3300 | |
| PFLU_3300_int_R | gcatcgcctgggtcatcctc | | Reverse internal primer for JE4621∆PFLU_3300 | |  |
| oPNL1658_PFLU_3302_UpF | tcaggaaacagctatgacatgattacgaattcacgctcttgttctggtgt | | Forward primer for PFLU_3302 upstream homology arm | |  |
| oPNL1659_PFLU_3302_UpR | acatcgaacatgctagaactcggcgatcagtcctgataga | | Reverse primer for PFLU_3302 upstream homology arm | |  |
| oPNL1660_PFLU3302_DnF | gaaggtctatcaggactgatcgccgagttctagcatgttcgatg | | Forward primer for PFLU_3302 downstream homology arm | |  |
| oPNL1661_PFLU_3302_DnR | ttgtaaaacgacggccagtgccaagctttggtttgccagtgttcac | | Reverse primer for PFLU_3302 downstream homology arm | |  |
| oPNL1662_PFLU3302_ScrF | atctcaaccttgactgtggtc | | Forward flanking primer for JE4621∆PFLU_3302 | |  |
| oPNL1663_PFLU3302_ScrR | gattcaggcagtaacaggatc | | Reverse flanking primer for JE4621∆PFLU_3302 | |  |
| oPNL1669_PFLU3302_INT_Rev | aacaaccttccacgtctg | | Forward internal primer for JE4621∆PFLU_3302 | |  |
| oPNL1557_PFLU3303_scrF_2 | tcgcgcttgttgatacg | | Reverse internal primer for JE4621∆PFLU_3302 | |  |
| oPNL1511 (PFLU3303-3304_UpF) | ggaaacagctatgacatgattacgaattcgccgctgcggtccggtgac | | Forward primer for PFLU_3303 upstream homology arm | |  |
| oPNL1664_PFLU3303_UP_rev | tgctcaattggctgcacaagttctaggggcaaacctcattgttg | | Reverse primer for PFLU_3303 upstream homology arm | |  |
| oPNL1665_PFLU3303_DN_FWD | ccataaaaacaacaatgaggtttgcccctagaacttgtgcagccaa | | Forward primer for PFLU_3303 downstream homology arm | |  |
| oPNL1666_PFLU3303_DN_REV | taaaacgacggccagtgccaagcttattgcccaaacaggatctg | | Reverse primer for PFLU_3303 downstream homology arm | |  |
| oPNL1516 (PFLU3303-3304_ScF) | tcggcggcacgcttgtcg | | Forward flanking primer for JE4621∆PFLU_3303 | |  |
| oPNL1667_PFLU3303_Scr_Rev | ggggtcttgaacaacaa | | Reverse flanking primer for JE4621∆PFLU_3303 | |  |
| oPL1559_PFLU3303_inF_2 | tgaacactggcaaacca | | Forward internal primer for JE4621∆PFLU_3303 | |  |
| oPNL1668_PFLU3303_Int_R | tacacgcacaacaccact | | Reverse internal primer for JE4621∆PFLU_3303 | |  |
| oPNL1539 PFLU3311_UpF | caggaaacagctatgacatgattacgaattcgtccagcccgcgcttgcctt | | Forward primer for PFLU_3311 upstream homology arm | |  |
| oPNL1485 (PFLU3311 Up_R) | gcaagactcttattttttgacgaaggggataaccctggaaatgac | | Reverse primer for PFLU_3311 upstream homology arm | |  |
| oPNL1486 (PFLU3311 Dn_F) | gagtcatttccagggttatccccttcgtcaaaaaataagag | | Forward primer for PFLU_3311 downstream homology arm | |  |
| oPNL1540 PFLU3311_DnR | tgtaaaacgacggccagtgccaagctttcgagggtgtaatccaggtt | | Reverse primer for PFLU_3311 downstream homology arm | |  |
| oPNL1481 (PFLU3311 Ext_F) | cacagcagttggtcaggaac | | Forward flanking primer for JE4621∆PFLU_3311 | |  |
| oPNL1541 PFLU3311_ScR | ggcgtcggggaagcgtg | | Reverse flanking primer for JE4621∆PFLU_3311 | |  |
| oPNL1479 (PFLU3311 Int_F) | atcatgccgtcattggccag | | Forward internal primer for JE4621∆PFLU_3311 | |  |
| oPNL1480 (PFLU3311 Int_R) | agcatgcgcacgctgtcgg | | Reverse internal primer for JE4621∆PFLU_3311 | |  |

| **Supplementary Table S3. Orthologs of SBW25 genes in other Pseudomonads** | | | | |  |  |  |  |  |  |  |  |  |  |
| --- | --- | --- | --- | --- | --- | --- | --- | --- | --- | --- | --- | --- | --- | --- |
|  |  |  | **KT2440** | | | | **TBS10** | | | | **TBS28** | | | |
|  | **SBW25 locus** | **product** | **locus** | **% identity** | **% positive** | **% gaps** | **locus** | **% identity** | **% positive** | **% gaps** | **locus** | **% identity** | **% positive** | **% gaps** |
| ech operon | PFLU3296 | acyl-CoA dehydrogenase | PP3354 | 66 | 74 | 2 | --- | --- | --- | --- | --- | --- | --- | --- |
|  | PFLU3297 | thiolase family protein | PP3355 | 75 | 81 | 0 | --- | --- | --- | --- | --- | --- | --- | --- |
|  | PFLU3298 | feruloyl-CoA synthase | PP3356 | 77 | 85 | 0 | PFR10_01918 | 85 | 92 | 0 | PFA28_01839 | 86 | 92 | 0 |
|  | PFLU3299 | aldehyde dehydrogenase | PP3357 | 87 | 92 | 0 | PFR10_01919 | 92 | 94 | 0 | PFA28_01838 | 91 | 94 | 0 |
|  | PFLU3300 | p-hydroxycinnamoyl CoA hydratase/lyase | PP3358 | 93 | 96 | 0 | PFR10_01920 | 99 | 100 | 0 | PFA28_01837 | 98 | 99 | 0 |
| ech regulator | PFLU3301 | MarR family transcriptional regulator | PP3359 | 80 | 87 | 0 | PFR10_01921 | 80 | 88 | 0 | PFA28_01836 | 83 | 90 | 0 |
| conserved PP transporters | PFLU3302 | OprD family porin | --- | --- | --- | --- | PFR10_01922 | 82 | 90 | 0 | PFA28_01835 | 80 | 89 | 0 |
|  | PFLU3303 | 3-(3-hydroxy-phenyl)propionate transporter MhpT | PP3349 | 58 | 72 | 0 | PFR10_01923 | 82 | 89 | 0 | PFA28_01834 | 85 | 90 | 0 |
| uncharacterized MHPP-upregulated gene cluster | PFLU3304 | hypothetical protein | --- | --- | --- | --- | --- | --- | --- | --- | --- | --- | --- | --- |
|  | PFLU3305 | coniferyl alcohol dehydrogenase | --- | --- | --- | --- | PFR10_01928 | 69 | 78 | 0 | PFA28_01987 | 71 | 81 | 0 |
|  | PFLU3306 | benzaldehyde dehydrogenase | --- | --- | --- | --- | PFR10_01929 | 86 | 92 | 0 | --- | --- | --- | --- |
|  | PFLU3307 | sigma-54-dependent Fis family transcriptional regulator | --- | --- | --- | --- | PFR10_01930 | 79 | 85 | 0 | PFA28_01986 | 54 | 69 | 4 |
|  | PFLU3308 | TetR/AcrR family transcriptional regulator | --- | --- | --- | --- | --- | --- | --- | --- | --- | --- | --- | --- |
|  | PFLU3309 | polyamine ABC transporter substrate-binding protein | PP5341 | 55 | 73 | 0 | PFR10_04530 | 54 | 73 | 0 | PFA28_02290 | 82 | 90 | 0 |
|  | PFLU3310 | hypothetical protein | --- | --- | --- | --- | --- | --- | --- | --- | PFA28_02291 | 82 | 88 | 0 |
|  | PFLU3311 | serine hydrolase | --- | --- | --- | --- | --- | --- | --- | --- | PFA28_02292 | 81 | 87 | 0 |
| Results from BLASTp pairwise alignment of SBW25 proteins with most highly similar proteins in the indicated organism. No value is listed if the most similar protein has <50% identity and <90% query coverage. | | | | | | | | | | | | | | |

| **Supplementary Table S4. Sequences of functionally verified serine hydrolases.** | |
| --- | --- |
| **Protein** | **Protein Sequence** |
| Pme from Pseudomonas fluorescens SBW25 (PFLU3311) | MGQNIMPSLASLYVETNESSTQPRLAPLLMQGFPAGPKYRVTWHNWMRPPFNQWGFRNLA  RLRPSIDVRAGAGPAGPLNTVSQALDALYFDSECGLSVSVIEHLLASQTDAFLVMQGDTV  LFERYFNGQRPCDRHIMFSVTKSLVGTLGEELVTRGVLNPELPAGYYVPELVGSAFGDAT  VRQLFDMAVGIDYSEVYDDPNSESSQYGYACGFQPALAQYAQFESLYQYLPSLKKRGVHG  GFFHYVTATTEALAWVMERASGSACSELLEGIWQQLGCDRDGYFIADPWGRNVAGAGFSA  TLRDMARFGRLLANNGRQDGVELLSPETVARITAGADPAVYAQNAEFSHWTPGASYRSQW  YVFNDHSQALMAGGIHGQYLFIDKPSGVVIVKQSSLNEAVSPFDTDSVRMLRAIAAHLSH |
| Pme from Pseudomonas sp. TBS28 (PFA28_02292) | MSQNAVPSLASLYVEAIESSDPRSTSSFMQGFPPEPQRRVSWHNWMRAPFNQWGFRNLAR  LRPSIDVQAGAAPVSPLQQAPQPLDQLHFNSECGLSISVIEHLLASQTDAFLVMQGDTVL  YERYFNGQRPQDRHIMFSVTKSLIGTLGEQLVCEGLLDTALPAAHYVPELAGSAFADATV  RQLFDMAVGIDYSEVYEDPDSESSQYGYACGFQPAPVQYGQFESLYEYLPSLRKRGSHGG  FFHYVTATTEALAWVMERACGRACHELLQDIWSQLGCERDGYFMADPWGRNVAGAGFSAT  LRDMARFGRLLANEGRHAGRQLLSSEAIAGILAGADPAVYATSPDFSAWTPGASYRSQWY  VFNDHSQALMAGGIHGQYLFVDKPSGVVIVKQSSLSEAVSPFDGDSVRMLRAIAAHLTR |
| NylB from Paenarthobacter ureafaciens KI72  (previously Arthobacter sp. KI72) | MNARSTGQHPARYPGAAAGEPTLDSWQEAPHNRWAFARLGELLPTAAVSRRDPATPAEPV  VRLDALATRLPDLEQRLEETCTDAFLVLRGSEVLAEYYRAGFAPDDRHLLMSVSKSLCGT  VVGALIDEGRIDPAQPVTEYVPELAGSVYDGPSVLQVLDMQISIDYNEDYVDPASEVQTH  DRSAGWRTRRDGDPADTYEFLTTLRGDGGTGEFQYCSANTDVLAWIVERVTGLRYVEALS  TYLWAKLDADRDATITVDQTGFGFANGGVSCTARDLARVGRMMLDGGVAPGGRVVSQGWV  ESVLAGGSREAMTDEGFTSAFPEGSYTRQWWCTGNERGNVSGIGIHGQNLWLDPRTDSVI  VKLSSWPDPDTRHWHGLQSGILLDVSRALDAV |
| NylB from Pseudomonas sp. NK87 | MNTVPPFRDPTVPGNSHIPRQDWDRAPWNRWTFQHVRELLPTTKVWRGTGPASPLPVDLR  DIDAVSFAAEGQSHTIAGFLETSYADGFLVLHGGKIVAERYLNGMAPHTQHLSQSVAKSV  VGTVAGILIDRGVVNPAALLTHYLPELEATAYRGATVQHVLDMTSGVVFDETYTALDSHM  AQLDVASGWKDSPNPDWPTHVWDLILSLKDLECPHGASFRYRSIETDVLAFVLQRAAAAP  LAELVSRELWAPMGAEEDAYFTVDTAGYALGDGGFNATLRDYARFALLHLRGGEIDGRRI  VSPGWIAATRFGADPALFGDIYREALPAGAYHNQFWIEDTARGAYMARGVFGQLIYIDPE  ADFAAVILSSWPEFVSTTRLRTALAAVRAVREALSA |

**References**

1. Kanagawa K, Oishi M, Negoro S *et al.* Characterization of the 6-aminohexanoate-dimer hydrolase from pseudomonas sp nk87. *Journal of General Microbiology*. 1993;**139**:787-95 https://doi.org/Doi 10.1099/00221287-139-4-787

2. Kinoshita S, Terada T, Taniguchi T *et al.* Purification and characterization of 6-aminohexanoic-acid-oligomer hydrolase of flavobacterium sp. Ki72. *Eur J Biochem*. 1981;**116**:547-51 https://doi.org/10.1111/j.1432-1033.1981.tb05371.x

3. Bailey MJ, Lilley AK, Thompson IP *et al.* Site directed chromosomal marking of a fluorescent pseudomonad isolated from the phytosphere of sugar beet; stability and potential for marker gene transfer. *Mol Ecol*. 1995;**4**:755-63 https://doi.org/10.1111/j.1365-294x.1995.tb00276.x

4. Bagdasarian M, Lurz R, Ruckert B *et al.* Specific-purpose plasmid cloning vectors. Ii. Broad host range, high copy number, rsf1010-derived vectors, and a host-vector system for gene cloning in pseudomonas. *Gene*. 1981;**16**:237-47 https://doi.org/10.1016/0378-1119(81)90080-9

5. Elmore JR, Dexter GN, Baldino H *et al.* High-throughput genetic engineering of nonmodel and undomesticated bacteria via iterative site-specific genome integration. *Sci Adv*. 2023;**9**:eade1285 https://doi.org/10.1126/sciadv.ade1285

6. Paulsen IT, Press CM, Ravel J *et al.* Complete genome sequence of the plant commensal pseudomonas fluorescens pf-5. *Nat Biotechnol*. 2005;**23**:873-8 https://doi.org/10.1038/nbt1110

7. Vermeiren H, Willems A, Schoofs G *et al.* The rice inoculant strain alcaligenes faecalis a15 is a nitrogen-fixing pseudomonas stutzeri. *Syst Appl Microbiol*. 1999;**22**:215-24 https://doi.org/10.1016/S0723-2020(99)80068-X

8. Elmore JR, Furches A, Wolff GN *et al.* Development of a high efficiency integration system and promoter library for rapid modification of pseudomonas putida kt2440. *Metab Eng Commun*. 2017;**5**:1-8 https://doi.org/10.1016/j.meteno.2017.04.001

9. Jayakody LN, Johnson CW, Whitham JM *et al.* Thermochemical wastewater valorization via enhanced microbial toxicity tolerance. *Energy & Environmental Science*. 2018;**11**:1625-38 https://doi.org/10.1039/c8ee00460a

10. Elmore JR, Dexter GN, Salvachua D *et al.* Production of itaconic acid from alkali pretreated lignin by dynamic two stage bioconversion. *Nature Communications*. 2021;**12** https://doi.org/ARTN 2261

10.1038/s41467-021-22556-8
